# Development and Validation of an Italian Adaptation of the Psychosocial Aspects of Hereditary Cancer Questionnaire

**DOI:** 10.3389/fpsyg.2021.697300

**Published:** 2021-07-20

**Authors:** Marzena Franiuk, Elena Molinari, Linda Battistuzzi, Elisabetta Razzaboni, Elisabetta De Matteis, Daniela Turchetti, Lea Godino, Carlo Chiorri, Liliana Varesco

**Affiliations:** ^1^Unit of Hereditary Cancer, IRCCS Ospedale Policlinico San Martino, Genova, Italy; ^2^UOSD Physical Medicine and Rehabilitation, IRCCS Giannina Gaslini Institute, Genova, Italy; ^3^Department of Informatics, Bioengineering, Robotics and Systems Engineering, University of Genoa, Genoa, Italy; ^4^Servizio di Psicologia Ospedaliera, AOU di Modena, Modena, Italy; ^5^Department of Oncology, Vito Fazzi Hospital, Lecce, Italy; ^6^Department of Medical and Surgical Sciences, Center for Studies on Hereditary Cancer and U.O. Genetica Medica, IRCCS Azienda Ospedaliero-Universitaria di Bologna, University of Bologna, Bologna, Italy; ^7^Department of Educational Sciences, University of Genova, Genova, Italy

**Keywords:** genetic counseling, genetic testing, psychological assessment, cancer, hereditary cancer

## Abstract

Individuals that attend cancer genetic counseling may experience test-related psychosocial problems that deserve clinical attention. In order to provide a reliable and valid first-line screening tool for these issues, Eijzenga and coworkers developed the Psychosocial Aspects of Hereditary Cancer (PAHC) questionnaire. The aim of this work was to develop an Italian adaptation of the PAHC (I-PACH). This prospective multicenter observational study included three stages: (1) development of a provisional version of the I-PAHC; (2) pilot studies aimed at testing item readability and revising the questionnaire; and (3) a main study aimed at testing the reliability and validity of the final version of the I-PAHC with the administration of a battery comprising measures of depression, anxiety, worry, stress, and life problems to 271 counselees from four cancer genetic clinics. Adapting the original PAHC to the Italian context involved adding two further domains and expanding the emotions domain to include positive emotions. While most of the items were found to be easy to understand and score, some required revision to improve comprehensibility; others were considered irrelevant or redundant and therefore deleted. The final version showed adequate reliability and validity. The I-PAHC provides comprehensive content coverage of cancer genetic-specific psychosocial problems, is well accepted by counselees, and can be considered a sound assessment tool for psychosocial issues related to cancer genetic counseling and risk assessment in Italy.

## Introduction

Genetic counseling has been defined as “the process of helping people understand and adapt to the medical, psychological and familial implications of genetic contributions to disease” (Resta et al., 2006, p. 77). This process includes as: the interpretation of family and medical histories in order to assess the chance of disease occurrence or recurrence; education about inheritance, testing, management, prevention, resources, and research; and counseling to promote informed choices and adaptation to the risk or condition (Resta et al., 2006). This definition emphasizes that genetic counseling is a communication process and that helping people to adjust to the medical, familial, and psychological implications of genetic information requires counseling skills. Approximately 10–25% of counselees experience heightened levels of distress during and/or after the genetic counseling process (Pasacreta, 2003; Voorwinden and Jaspers, 2016), but genetic counselors often fail to recognize and address these issues, since they tend to be more focused on gathering and giving medical information (Meiser et al., 2008), and report a lack of appropriate tools assessing the specific psychosocial problems and distress levels experienced by counselees.

Some attempts have already been made to address this need, such as the Multidimensional Impact of Cancer Risk Assessment (Cella et al., 2002), the Psychological Adaptation to Genetic Information Scale (Read et al., 2005), and the Genetic Counseling Outcome Scale (McAllister et al., 2011). These measures focus on the psychological impact of and adaptation to genetic test results, but although they can provide important information *after* the genetic counseling process is completed, they do not cover other relevant issues, such as worries about undergoing cancer risk assessment. Two other questionnaires assess counselees’ concerns *during* genetic counseling: the Genetic Risk Assessment Coping Evaluation (GRACE; Bennett et al., 2012) and the Psychosocial Aspects of Genetic Counselling (PAHC; Eijzenga et al., 2014b). By breaking down an ongoing stressful event into its component parts, the GRACE helps identify the ways in which individuals choose to cope with these demands; however, it does not assess other important areas, such as the burden of having (or having had) cancer or experiencing cancer in the family. With its 26 items covering six problem domains (genetics, practical issues, family, living with cancer, emotions, and children), the PAHC instead appears to be the most comprehensive measure of the psychosocial aspects of cancer genetic counseling among those currently available and has proven to be useful in improving counselor-counselee communication and decreasing counselee distress (Eijzenga et al., 2014b).

In Italy, the professional figure of the genetic counselor does not exist and counseling is carried out by medical geneticists, biologists, or oncologists. During their training, none of these healthcare professionals receive a solid grounding in the principles and practice of counseling, nor is such grounding a formal requirement to practice genetic counseling in Italy. Furthermore, no evidence-based guidelines are usually offered on psychological support to counselees, even if international recommendations have been published (Kääriäinen et al., 2010). However, the possible negative consequences of oncogenetic counseling on psychological wellbeing of Italian counselees and the need of a psycho-oncologist in a multidisciplinary team have been pointed out more than a decade ago (e.g., Condello et al., 2007; Caruso et al., 2008). Recent studies carried out in Italy have shown that ex-patients and affected patients, especially if females and younger, tend to report higher levels of anxiety, depression, negative mood, and genetic risk perception, and lower levels of quality of life and wellbeing than healthy counselees, and thus may be at risk of psychological discomfort during the counseling process (e.g., Cicero et al., 2017; Mella et al., 2017; Di Mattei et al., 2018; Ballatore et al., 2020). Therefore, identifying counselees’ personal and psychosocial needs before and after genetic testing could help Italian practitioners target those individuals who are most likely to seek and to use psychological service (Maheu et al., 2014).

In order to provide a tool that will help address these issues in Italian cancer genetic services, and given the proven efficacy of the PAHC in facilitating genetic counselors in recognizing and discussing counselees’ psychosocial problems and reducing their distress levels (Eijzenga et al., 2014a,b,c), we developed and validated an Italian adaptation of the PAHC.

## Materials and Methods

### Development of the Italian Version of the PAHC Questionnaire

We originally planned to simply translate PACH into Italian using the common translation-back translation method (Behling and Law, 2000). However, when reviewing item content, we found that some items did not reflect common cancer genetic counseling practices in Italy and that, according to our collective understanding of and experience with psycho-social issues reported by counselees in Italy, some apparently important domains were missing. We thus decided to develop an adaptation that maintained the original six problem domains of the PAHC but included two further domains: motivation to undergo the genetic test and perceived social support. Moreover, we expanded the emotions domain of the PACH to include positive emotions. The question “would you like to speak with a psychosocial worker in addition to the clinical geneticist/genetic counselor about these issues?” at the end of each domain was maintained. The result was the provisional 48-item Italian PAHC (I-PAHC).

The answer scale was the same as that of the original PAHC, namely: The person completing the questionnaire was asked to indicate the level of perceived worry in relation to the situation described in the item and the intensity of the emotions experienced through a 4-point Likert-type scale (1 = “not at all;” 2 = “a little;” 3 = “quite a bit;” and 4 = “very much”).

### Pilot Studies on Item Readability

The provisional I-PAHC was administered to 17 counselees from the leading center in order to test the readability and understandability of each item. A licensed psychologist conducted a semi-structured interview and asked participants to rephrase items in their own words and to comment them. A detailed description of the procedure is provided in Section 1 of the Supplementary Material (SM). Overall, the results revealed that most participants perceived the questionnaire as useful and clear. However, a careful review of the transcripts by the study team identified three main issues: (a) some participants found some words difficult to understand; (b) many participants reported that some items were repetitive or redundant; and (c) many participants stated that it was difficult to answer using the 4-point Likert-type response scale and that they would have preferred more intermediate options. As a result, some items were reworded to improve their comprehensibility, those identified as redundant or irrelevant were removed, and the answer scale length was extended to seven points. The abridged version of I-PAHC thus comprised 31 items and was administered to 40 new participants, 10 from each study center, with the same procedure. This time, participants raised only minor issues that could be addressed without substantially changing the questionnaire. In general, they considered the length of the questionnaire adequate, the graphics pleasant, and the items easy to understand and to score. The final version of the I-PAHC is reported in the Appendix (see Supplementary Material) and was administered, together with a battery comprising measures of depression, anxiety, worry, stress, and life problems to 271 counselees from the four study centers, as described below.

### Main Study Participants

Participants were 271 counselees recruited from four Italian cancer genetic clinics: a Hereditary Cancer Unit from a Northern Italy hospital (154, 56.80%), a Unit of Medical Genetics from a Central Italy hospital (57, 21.00%), an Oncology Unit from a Southern Italy hospital (40, 14.80%), and an Oncogenetic Unit from another Central Italy Hospital (20, 7.40%). To qualify for the study counselees had to be 18 or older and had to have been referred for cancer genetic testing for hereditary breast/ovarian or colorectal cancer, regardless of whether they had been diagnosed with cancer or not. Table 1 shows the socio-demographic characteristics of the participants.

**Table 1:** Socio-demographic characteristics of the study sample (*n* = 271).

Variable	Statistic
Age (years; mean ± standard deviation, range)	52.02 ± 11.17 (19–78)
Gender (N, %)	
Female	249 (91.88%)
Male	19 (7.01%)
Not reported	3 (1.11%)
Education level (N, %)	
Primary school (5 years of education)	13 (4.80%)
Secondary school (8 years of education)	37 (13.65%)
High school (13 years of education)	140 (51.66%)
Bachelor degree (16 years of education)	14 (5.17%)
Master degree (18 years of education)	52 (19.19%)
Post-secondary degree (>20 years of education)	10 (3.69%)
Not reported	5 (1.85%)
Marital status	
Single	34 (12.55%)
Cohabitating	23 (8.49%)
Married	162 (59.78%)
Divorced	31 (11.44%)
Widow/widower	16 (5.90%)
Not reported	5 (1.85%)
Occupational status	
Unemployed	14 (5.17%)
Employee	129 (47.60%)
Self-employed	36 (13.28%)
Retired	44 (16.24%)
Housewife	36 (13.28%)
Student	6 (2.21%)
Not reported	6 (2.21%)
Children	
Yes	206 (76.01%)
No	60 (22.14%)
Not reported	5 (1.85%)
Current cancer diagnosis	
Yes	76 (28.04%)
No	191 (70.48%)
Not reported	4 (1.48%)
Former cancer diagnoses	
None	107 (39.48%)
One	109 (40.22%)
Two	29 (10.70%)
Three	6 (2.21%)
Not reported	20 (7.38%)

### Main Study Measures

The battery comprised a form for collecting background information and some psychological questionnaires.The form for collecting background information focused on educational levels, occupational, and marital status, whether participants had any children, and if so how many and their ages. Information about the type of cancer the participant had developed, if any, whether there had been previous cancers, and whether the participant was under medication was also collected.

#### The Italian Psychosocial Aspects of Genetic Counselling

As described above, see Appendix in the Supplementary Material.

#### The National Comprehensive Cancer Network Distress Thermometer

(DT; Psychosocial Distress Practice Guidelines Panel, 1999). The DT is a single item, 11-point rating scale graphically depicted as a thermometer that ranges from 0 (no distress) to 10 (extreme distress), through which patients can indicate their level of distress over the course of the week prior to the visit. The DT is complemented by a list of 40 problems (6 practicals, 4 family-related, 6 emotional, 1 spiritual/religious, and 23 physical) that map common issues related to the cancer experience (see Appendix).

#### State Anxiety Inventory-X3

The State Anxiety Inventory-X3 (STAI-X3) is a 10-item unidimensional measure of state anxiety. Participants are asked to rate on a 4-point, Likert-type, intensity scale how they feel “right now” with respect to some symptoms of anxiety (or lack thereof; e.g., being calm, tense, and preoccupied; Bertolotti et al., 1990).

#### Center for Epidemiological Studies Depression Scale-Short Form

The Center for Epidemiological Studies Depression Scale-Short Form (CES-D-SF) is a 10 item, shorter version of the original CES-D (Radloff, 1977) and includes questions that investigate whether respondents have experienced some of the most common depression symptoms (e.g., unhappiness, sadness, and loneliness) in the last week on a 4-point, Likert-type, frequency scale (Kohout et al., 1993, Italian version in Fava, 1983).

#### Penn State Worry Questionnaire

The Penn State Worry Questionnaire (PSWQ) is a 16-item questionnaire that assesses trait worry, i.e., “a chain of thoughts and images, negatively affect-laden and relatively uncontrollable; it represents an attempt to engage in mental problem-solving on an issue whose outcome is uncertain but contains the possibility of one or more negative outcomes” (Borkovec et al., 1983, p. 10). The items operationalize the generality, excessiveness, and uncontrollability of such trait and respondents are asked to report the extent to which each item is typical of them on a 5-point Likert-type scale (Meyer et al., 1990, Italian version in Morani et al., 1999).

### Main Study Procedure

Participants completed the questionnaire during or after the genetic counseling session. The order of the questionnaires within each battery varied according to the balanced Latin square design to minimize order and sequence effects. The mean administration time of the battery was 18.21 minutes (*SD* = 6.20, range 5–35). All participants provided written informed consent and were treated according to the principles laid out in the Declaration of Helsinki (World Medical Association, 2013). The study was approved by the Ethical Committee of the study leading center.

## Results

Given the nesting in participants into centers, we used a multilevel approach (Taplin et al., 2012) to data analysis. Full-information maximum likelihood methods were used to handle missing data (Collins et al., 2001).

Administration time increased with age and a current diagnosis of cancer (Pearson’s *r* = 0.39, *p* < 0.001 and *r* = 0.21, *p* < 0.001, respectively), decreased with education level (Spearman’s *ρ* = −0.21, *p* < 0.001), and was not associated with the number of former diagnoses (*ρ* = 0.11, *p* = 0.129).

Detailed descriptive statistics for the I-PAHC items are reported in Section 2 of the Supplementary Material. The results showed that participants reported a relatively high motivation to undergo the genetic test (mean score on I-PACH1: 6.15 ± 1.28). Nearly three out of five participants (64.88%) sought advice about this from a specialist, mainly their oncologist (44.50%), general practitioner (18.92%), and gynecologist (15.83%). Almost all participants found the advice given useful (92.48%).

For the first three scales (Hereditary predisposition [items 3–8], Family and social environment [items 9–13], and Children [items 15b–18]), we performed exploratory factor analyses and item analyses to investigate their unidimensionality and reliability. In all three cases, the dimensionality analyses (scree-plot, parallel analysis, and minimum average partial correlation statistic) suggested that the optimal number of factors to be extracted was one (see description of the analyses and detailed results in Section 3 of the Supplementary Material). The proportion of variance accounted for by the single factor was 0.40, 0.37, and 0.37, respectively, and the factor loadings ranged from 0.39 to 0.70. Corrected item-total correlations ranged from 0.33 to 0.68, squared multiple correlations ranged from 0.12 to 0.49, and in no case the alpha without the item statistic exceeded the alpha coefficient (0.79, 0.73, and 0.71, respectively; see description of the analyses and detailed results in Section 3 of the Supplementary Material). Taken together, these results suggest that the three scales considered had adequate levels of unidimensionality and reliability. The correlations of the scale scores with the wish to speak with a psychologist about these issues were 0.18 (*p* = 0.002), 0.22 (*p* < 0.001), and 0.24 (*p* < 0.001), respectively, indicating that the higher the worry for the issues assessed by the scale, the higher the need to speak with a psychologist.

For the Perceived Support scale (items 19a–21b), we could not perform the same analyses, as items depended on whether the participant had spoken about the genetic test with their partner, family, and/or their friends. Hence, we simply computed correlations between the answers to the items and the wish to speak with a psychologist about these issues. The only significant correlation was the one of I-PACH20b (*r* = −0.20, *p* = 0.004) that indicated that the higher the perceived support by family, the lower the need to speak with a psychologist (see Table SM2.21PSY4a in the Supplementary Material for more details).

For the Emotions scale (items 22–29), we expected a two-factor structure, since we revised some of the negative affect items of the original PAHC and added positive affect items, and the results supported the prediction (see description of the analyses and detailed results in Section 3 of the Supplementary Material). The proportion of variance accounted for by the solution was 0.61, item loadings ranged from 0.53 to 0.93, and the two factors were only moderately correlated (*r* = −0.35, *p* < 0.001), suggesting that they assess two distinct emotional constructs. Corrected item-total correlations ranged from 0.51 to 0.82, squared multiple correlations ranged from 0.27 to 0.69, and in no case the alpha without the item statistic exceeded the alpha coefficient (0.87 and 0.83 for the Negative and Positive Affect scales, respectively; see description of the analyses and detailed results in Section 3 of the Supplementary Material). The correlations of the scale scores with the wish to speak with a psychologist about these issues for the Negative and Positive Affect scales were 0.25 (*p* = 0.002) and −0.13 (*p* = 0.032), respectively, indicating that the higher the negative affect and the lower the positive affect, the higher the need to speak with a psychologist.

For the scale Illness experience scale items (30a–33b), we adopted the same strategy as for the Perceived Support scale. In this case, only item 32 showed a marginally significant correlation (*r* = 0.12, *p* = 0.052) with the wish to speak with a psychologist about these issues, indicating that the higher the frequency of thinking about developing cancer, the higher the need to speak with a psychologist (see Table S2.33PSY5a in the Supplementary Material for more details).

Finally, we computed correlations between I-PACH scores and the socio-demographic variables and the other psychological measures. Given the large number of coefficients computed, the inflation of the Type I error rate due to multiple comparisons would have increased the probability of wrongly rejecting the null hypothesis of no association far beyond the common accepted threshold of 0.05. In order to address this issue, we lowered the significance level to 0.001 and, given the sample size, we computed the two-tailed critical correlation value, which was 0.20.

Motivation to undergo the genetic test and having or not sought advice from a specialist were not predictive of any criterion. For the socio-demographic variables, we found that scores on the Hereditary predisposition, Children, and Negative emotions scales decreased with age, while the score on the Children scale was higher in married or cohabiting participants (see SM5.1 in Supplementary Material). The score in the perceived support from friends also decreased with age, and among retired participants. As expected, the emotional burden from current cancer was highly correlated with actually having cancer, while the burden from past cancer was highly correlated with having had cancer in the past (Supplementary Table SM5.1).

The correlations with the psychological constructs are reported in Table 2. The multi-item scales of the I-PAHC showed the expected pattern of moderate-to-high positive correlations with scores of stress, emotional problems, anxiety, depression, and worry implied by the specific item content – except for the Positive Emotions scale, in which correlations were negative. While no significant correlation of I-PACH scores with levels of practical and spiritual problems was found, we observed negative correlations of others’ support items with the family-related problems scale and positive correlations of the Negative Emotions scale and of the emotional burden of current cancer with physical problems. Lower perceived support from partner was associated with higher levels of family-related problems, while lower perceived support from family was associated with higher levels of depressive symptoms.

**Table 2:** Correlations of I-PACH scores with the other psychological measures.

I-PAHC variable	DT	Prac	Fam	Emo	Phys	Spir	STAI-X3	CES-D	PSWQ
I-PAHC1 – motivation	0.04	0.10	0.06	0.07	0.18	0.00	−0.10	0.02	0.01
I-PAHC2 – no advice from specialist	−0.09	−0.04	−0.03	−0.09	−0.02	−0.05	−0.16	−0.02	−0.05
I-PAHC2 – useful advice from specialist	0.00	0.02	−0.01	0.08	0.01	0.01	0.13	−0.02	0.03
I-PAHC2 – useless advice from specialist	**0.20**	0.04	0.08	0.02	0.02	0.09	0.06	0.09	0.04
Hereditary predisposition	**0.27**	0.04	0.10	**0.38**	0.15	0.08	**0.39**	**0.26**	**0.39**
Family and social relationships	0.19	0.01	0.10	0.20	0.10	0.03	**0.33**	**0.21**	**0.26**
Children	**0.28**	0.16	0.18	**0.28**	0.20	−0.02	**0.34**	**0.36**	**0.35**
I-PAHC19b – support from partner	0.03	−0.19	**−0.25**	−0.01	−0.06	−0.02	0.03	−0.12	−0.08
I-PAHC20b – support from family	−0.03	−0.13	−0.16	0.07	−0.11	−0.12	−0.03	**−0.21**	−0.11
I-PAHC21b – support from friends	0.12	−0.10	−0.08	0.03	−0.07	0.08	0.16	−0.10	−0.03
Negative Emotions	**0.41**	0.07	0.14	**0.50**	**0.28**	0.06	**0.64**	**0.39**	**0.37**
Positive Emotions	**−0.21**	−0.06	−0.09	**−0.24**	−0.06	−0.03	**−0.27**	**−0.26**	−0.19
I-PAHC30 – emotional burden current cancer	0.11	−0.04	−0.05	0.16	**0.22**	0.09	**0.23**	**0.23**	0.04
I-PAHC31 – emotional burden past cancer	0.06	−0.02	0.05	−0.01	0.06	0.02	−0.01	0.05	0.09
I-PAHC32 – worry for cancer	**0.29**	0.12	0.12	**0.27**	0.20	0.14	**0.29**	**0.32**	**0.34**
I-PAHC33 – impact of a loved one’s cancer	0.12	0.07	0.00	0.14	0.00	0.03	0.13	0.09	0.13

Bolded values are correlations larger than |0.20|.

DT, Distress Thermometer; Prac, practical problems; Fam, family-related problems; Emo, emotional problems; Phys, physical problems, Spir, spiritual problems; CES-D, Center for Epidemiological Studies Depression Scale-Short Form; STAI-X3, State Anxiety Inventory-X3; PSWQ, and Penn State Worry Questionnaire.

## Discussion

The aim of the studies described in this work was to develop an Italian adaptation (I-PAHC) of Eijzenga and coworkers’ Psychosocial Aspects of Hereditary Cancer questionnaire. We used a multistage, standardized, and structured procedure for developing the I-PAHC, and both healthcare professionals’ and patients’ inputs were used. This ensured adequate content and face validity, making the I-PAHC a useful tool for understanding how counselees cope with the main psychological issues relating to genetic assessment.

The need to adapt the content of the original questionnaire to Italian counselees and to the specific ways in which cancer genetic counseling is practiced in Italy led us to perform a substantial revision of the content and wording of some items and the development of two new scales (motivation to undergo the genetic test and perceived social support). It should be noted that such changes were made to address the needs reported by participants from the target population in the pilot studies in order to better tailor the questionnaire. While these changes have no implications in terms of the validity of the original PAHC, they confirm that ensuring cognitive and contextual equivalence between a source instrument and the target will often require adaptation rather than simple translation (Beaton et al., 2000).

The four multi-item domains of the I-PAHC (Hereditary predisposition, Family and social relationships, Children, and Emotions) had adequate levels of unidimensionality, reliability, and construct validity, consistent with previous studies, including the Italian ones, that found that the psychological issues elicited by undergoing genetic counseling are associated with psychological distress, emotional problems, worry, and symptoms of anxiety and depression. The single items also showed the expected pattern of correlations with the other psychological questionnaires. In general, I-PACH scores were negligibly associated with background characteristics, except for a tendency to decrease with age. While this latter result is consistent with findings by Ballatore et al. (2020), the former replicates those already reported by Mella et al. (2017).

Notably, all scores were significantly associated with a positive answer to the question about the wish to speak with a psychologist in addition to the clinical geneticist/genetic counselor, albeit weakly. This result can be due to statistical reasons (i.e., the inevitable attenuation of the size of the correlation when correlating a metric and a dichotomous variable; Nunnally, 1975) and/or because of the majority of counselees has enough psychological and social coping resources to face with genetic information. Around 40% of the counselees in this study answered “yes” to the question about the wish to undergo psychological counseling for the domains tapped into by the I-PAHC, with this percentage being as low as 28% when perceived support issues were concerned. This result is consistent with previous studies (see, e.g., Vos et al., 2013), in which between 25 and 39% of all counselees have reported the wish for additional psychological support, and 27% requested psychological help after genetic testing intake. These findings suggest that counselees’ requests for psychological help are related to negative emotion and/or psychopathological states, but also to their need for more information and clarification, and for support when communicating with relatives and making test-related decisions. Some counselees may have concerns about the test that impact on their wellbeing without reaching pathological levels, but little is currently known about the best practices to provide tailored psychosocial services to patients referred for genetic testing for cancer risk (Oliveri et al., 2018). Furthermore, the sign of the correlation was in the expected direction, i.e., higher scores predict a higher probability of answering “yes” to the question about psychological counseling. As a guideline for Italian practitioners, we suggest considering “critical” scores that are equal to or higher than the third quartile (Table 3).

**Table 3:** Descriptive statistics of scores in the I-PACH items and scales.

I-PAHC variable/scale	Min	Q1	Median	Q3	Max
I-PAHC1 – motivation^	7	7	7	5	1
Hereditary predisposition	6	23	28	34	42
Family and social relationships	5	14	20	25	35
Children	4	12	18	22	28
I-PAHC19b – support from partner	1	5	7	7	7
I-PAHC20b – support from family	1	6	7	7	7
I-PAHC21b – support from friends	1	4	6	7	7
Negative Emotions	4	8	12	19	28
Positive Emotions^	28	23	18	15	4
I-PAHC30 – emotional burden current cancer	1	4	5	7	7
I-PAHC31 – emotional burden past cancer	1	4	5	7	7
I-PAHC32 – worry for cancer	1	4	5	7	7
I-PAHC33 – impact of a loved one’s cancer	1	5	7	7	7

Min, minimum; Q1, first quartile; Q3, third quartile; Max, maximum; and ^scores on this scale have been reverse scored in order to facilitate interpretation (i.e., the higher the quartile and the more distressed the patient).

The main limitation of this study is the lack of probabilistic sampling, which implies that the results may lack full generalizability to the population of counselees, which should also be considered when referring to the data in Table 3. However, it should be noted that the sample size used here was double the one used in the seminal study of the original PAHC. An important issue to be addressed by future research is the sensitivity to change of I-PAHC scores, namely, whether the questionnaire can be used as an outcome measure to evaluate the efficacy of interventions aimed at reducing counselees’ distress. Although the I-PAHC was well accepted by participants, it is not clear whether and how its use can affect counselor-counselee communication. As pointed out by one reviewer, it requires a good cognitive level to be able to be completed independently. Hence, counselees with cognitive problems, low cultural levels, or the elderly may encounter difficulties in filling it in and might need an administration method other than self-report.

## Conclusion

The evidence presented here suggests that the I-PAHC questionnaire can be a useful screening tool for detecting psychosocial issues among individuals attending cancer genetic counseling and testing in Italy. The I-PAHC provides information about the main factors that contribute to the stress arising from the process of cancer genetic risk assessment and, differently from the generic Distress Thermometer, it allows breaking down the stressful experience into its relevant components, enabling the identification of the elements of the cancer genetic risk assessment process that cause the most distress, and ultimately facilitating the development of targeted and tailored psychological counseling interventions.

## Data Availability Statement

The original contributions presented in the study are included in the article/Supplementary Material, further inquiries can be directed to the corresponding author.

## Ethics Statement

The studies involving human participants were reviewed and approved by Regional Ethical Committee of San Martino Hospital, Genoa. The patients/participants provided their written informed consent to participate in this study. Written informed consent was obtained from the individual(s) for the publication of any potentially identifiable images or data included in this article.

## Author Contributions

MF: Conceptualization, Data curation, Investigation, Methodology, Project administration, Visualization, Writing - Original Draft, Writing - Review & Editing. EM: Conceptualization, Data curation, Methodology, Visualization, Writing - Original Draft, Writing - Review & Editing. LB: Conceptualization, Methodology, Writing - Review & Editing. ER: Conceptualization, Investigation, Methodology, Resources, Writing – Review & Editing. ED: Investigation, Resources. DT: Investigation, Resources. LG: Investigation, Resources. CC: Software, Validation, Formal analysis, Data curation, Visualization, Writing - Original Draft, Writing - Review & Editing. LV: Conceptualization, Data curation, Funding acquisition, Methodology, Project administration, Visualization, Writing - Review & Editing. All authors contributed to the article and approved the submitted version.

### Conflict of Interest

The authors declare that the research was conducted in the absence of any commercial or financial relationships that could be construed as a potential conflict of interest.
