# Supplementary material for: Development and Validation of an Italian Adaptation of the Psychosocial Aspects of Hereditary Cancer Questionnaire
Source: Front Psychol. 2021 Jul 20;12:697300. doi: 10.3389/fpsyg.2021.697300 (PMC8329440; doi:10.3389/fpsyg.2021.697300)
Supplement: Supplementary file 1 [file Table_1.DOCX]

***Supplementary material***

# **Appendix**

In the following pages, we report the Italian PAHC. In order to facilitate the understanding of the items to non-Italian speaking readers, we show the English translation in red text. Please note that this translation is intended only to convey the content of the item, and might not be suitable for clinical and professional use without a proper adaptation procedure (see, e.g., (Behling and Law, 2000). The authors are available to assist and help in this process.

**Questionario sugli Aspetti Psicologici legati al Test Genetico di predisposizione ai tumori**

**Psychological Aspects of Hereditary Cancer Genetic Test Questionnaire**

Gentile Signora/Signore, il presente questionario ha lo scopo di aiutare Lei e noi operatori a mettere in luce aspetti “non medici” collegati alla Sua decisione di effettuare o meno il test genetico. È quindi importante che Lei risponda sinceramente a tutte le domande: non esistono risposte giuste o sbagliate, ma si assicuri che ognuna delle Sue risposte rifletta il più accuratamente possibile le Sue opinioni e/o i Suoi stati d'animo. Il questionario verrà archiviato nella Sua cartella ambulatoriale e verrà trattato in conformità con le disposizioni vigenti in materia di privacy (art. 13 del D. Lgs 196/2003).

Dear Madam/Sir,

The purpose of this questionnaire is to help us better understand your decision to have or not have the genetic test you have been offered. Please answer all the questions honestly: there are no right or wrong answers, but please make sure that each of your answers reflects your opinions and/or emotions as accurately as possible. The questionnaire will be filed in your outpatient folder and will be treated in accordance with current privacy law (art.13 of Legislative Decree 196/2003)

**Effettuare o meno il test genetico**

**Having the test or not**

| 1. Quanto si sente motivata/o ad effettuare il test genetico?  How much are you motivated to having the test? | \| Per niente  Not at all \| \| Mediamente  Somewhat \| \| \| Molto  Very \| \| \| --- \| --- \| --- \| --- \| --- \| --- \| --- \| \| ❑^1^ \| ❑^2^ \| ❑^3^ \| ❑^4^ \| ❑^5^ \| ❑^6^ \| ❑^7^ \| \|  \|  \|  \|  \|  \|  \|  \| |
| --- | --- | --- | --- | --- | --- | --- | --- | --- | --- | --- | --- | --- | --- | --- | --- | --- | --- | --- | --- | --- | --- | --- |
| 2. Rispetto alla decisione di fare o meno il test genetico, si è consultata/o con il medico di famiglia o con uno specialista?  Have you discussed your decision to have the test or not with your family doctor or a specialist physician? | \| No \| \| Sì, ed è stato utile  Yes, and it was helpful \| \| \| Sì, ma non è stato utile  Yes, but it was not helpful \| \| \| --- \| --- \| --- \| --- \| --- \| --- \| --- \| \| ❑^1^ \|  \|  \| ❑^2^ \|  \|  \| ❑^3^ \| |

2a. Se desidera, specifichi con quali professionisti si è confrontata/o:

Please report any healthcare professional you consulted

genetista geneticist

medico di famiglia family doctor

ginecologo gynecologist

oncologo oncologist

infermiere nurse

 chirurgo surgeon

psicologo psychologist

altro _other _____________________________________________________________________

**Predisposizione ereditaria**

**Hereditary predisposition**

| 3. Quanto è preoccupata/o della possibilità di essere portatrice/portatore di una mutazione genetica?  Are you worried about the chance of being a carrier of a genetic mutation? | \| Per niente  Not at all \| \| Mediamente  Somewhat \| \| \| Molto  Very \| \| \| --- \| --- \| --- \| --- \| --- \| --- \| --- \| \| ❑^1^ \| ❑^2^ \| ❑^3^ \| ❑^4^ \| ❑^5^ \| ❑^6^ \| ❑^7^ \| |  |  |
| --- | --- | --- | --- | --- | --- | --- | --- | --- | --- | --- | --- | --- | --- | --- | --- | --- | --- |
| 4. Quanto la preoccupano le possibili conseguenze dell’eventuale test genetico sulla Sua vita?  Are you worried about the impact the genetic test may have on your life? | \| Per niente  Not at all \| \| Mediamente  Somewhat \| \| \| Molto  Very \| \| \| --- \| --- \| --- \| --- \| --- \| --- \| --- \| \| ❑^1^ \| ❑^2^ \| ❑^3^ \| ❑^4^ \| ❑^5^ \| ❑^6^ \| ❑^7^ \| |  |  |
| 4a. Può indicare quali conseguenze la preoccupano di più?  Please list the consequences that worry you the most | | |  |
|  | | |  |
|  | | |  |
|  | | |  |
|  | | |  |
| 5. Quanto La preoccupa la possibilità di doversi sottoporre a sorveglianza intensificata (controlli medici più frequenti)?  Are you worried about having to have intensified screening (more frequent appointments)? | \| Per niente  Not at all \| \| Mediamente  Somewhat \| \| \| Molto  Very \| \| \| --- \| --- \| --- \| --- \| --- \| --- \| --- \| \| ❑^1^ \| ❑^2^ \| ❑^3^ \| ❑^4^ \| ❑^5^ \| ❑^6^ \| ❑^7^ \| |  |  |
| 6. Quanto La preoccupa la possibilità di sottoporsi a interventi chirurgici per ridurre il rischio di tumore?  Are you worried about having to have cancer risk reducing surgery? | \| Per niente  Not at all \| \| Mediamente  Somewhat \| \| \| Molto  Very \| \| \| --- \| --- \| --- \| --- \| --- \| --- \| --- \| \| ❑^1^ \| ❑^2^ \| ❑^3^ \| ❑^4^ \| ❑^5^ \| ❑^6^ \| ❑^7^ \| |  |  |
| 7. Quanto La preoccupa l'impatto che la Sua eventuale predisposizione ereditaria potrebbe avere sui Suoi progetti di vita?  Are you worried about the impact that hereditary susceptibility to cancer could have on your life plans? | \| Per niente  Not at all \| \| Mediamente  Somewhat \| \| \| Molto  Very \| \| \| --- \| --- \| --- \| --- \| --- \| --- \| --- \| \| ❑^1^ \| ❑^2^ \| ❑^3^ \| ❑^4^ \| ❑^5^ \| ❑^6^ \| ❑^7^ \| |  |  |
| 8. Quanto La preoccupa il fatto che la medicina abbia ancora incertezze in questo campo (es. sul significato di alcune mutazioni, su quale sia il modo migliore per affrontare il rischio di tumore etc.)  Are you worried about the fact that doctors still do not fully understand hereditary susceptibility to cancer (e.g., what certain mutations mean or how best to manage hereditary cancer risk)? | \| Per niente  Not at all \| \| Mediamente  Somewhat \| \| \| Molto  Very \| \| \| --- \| --- \| --- \| --- \| --- \| --- \| --- \| \| ❑^1^ \| ❑^2^ \| ❑^3^ \| ❑^4^ \| ❑^5^ \| ❑^6^ \| ❑^7^ \| |  |  |
| *Vorrebbe parlare di questi argomenti con uno psicologo, oltre che con il genetista? No* ❑^0^ *Sì* ❑^1^  **Would you like to talk to a psychologist in addition to the clinical geneticist about these issues?** *No* ❑^0^ *Yes* ❑^1^ | | | |
|  |  |  |  |

**Famiglia e relazioni sociali**

**Family and social environment**

| 9. Quanto La preoccupa dover comunicare ai Suoi famigliari l’esito del Suo eventuale test genetico?  Are you worried about sharing the results of your genetic test result with your family? | \| Per niente  Not at all \| \| Mediamente  Somewhat \| \| \| Molto  Very \| \| \| --- \| --- \| --- \| --- \| --- \| --- \| --- \| \| ❑^1^ \| ❑^2^ \| ❑^3^ \| ❑^4^ \| ❑^5^ \| ❑^6^ \| ❑^7^ \| |
| --- | --- | --- | --- | --- | --- | --- | --- | --- | --- | --- | --- | --- | --- | --- | --- |
| 10. La preoccupa dover eventualmente comunicare ai Suoi famigliari che è utile anche per loro sottoporsi al test genetico?  Are you worried about informing your relatives that they could benefit from having the genetic test? | \| Per niente  Not at all \| \| Mediamente  Somewhat \| \| \| Molto  Very \| \| \| --- \| --- \| --- \| --- \| --- \| --- \| --- \| \| ❑^1^ \| ❑^2^ \| ❑^3^ \| ❑^4^ \| ❑^5^ \| ❑^6^ \| ❑^7^ \| |
| 11. Le capita di pensare con preoccupazione alla possibilità che un Suo famigliare sviluppi un tumore?  Are you worried about the chance that a family member gets cancer? | \| Per niente  Not at all \| \| Mediamente  Somewhat \| \| \| Molto  Very \| \| \| --- \| --- \| --- \| --- \| --- \| --- \| --- \| \| ❑^1^ \| ❑^2^ \| ❑^3^ \| ❑^4^ \| ❑^5^ \| ❑^6^ \| ❑^7^ \| |
| 12. Quanto sente il peso della responsabilità nei confronti dei membri della Sua famiglia riguardo alla decisione di fare o di non fare il test genetico?  About your decision to have the test or not, how much do you feel the responsibility towards your family members? | \| Per niente  Not at all \| \| Mediamente  Somewhat \| \| \| Molto  Very \| \| \| --- \| --- \| --- \| --- \| --- \| --- \| --- \| \| ❑^1^ \| ❑^2^ \| ❑^3^ \| ❑^4^ \| ❑^5^ \| ❑^6^ \| ❑^7^ \| |
| 13. Quanto La preoccupa il fatto di poter essere trattata/o diversamente rispetto a prima dalle persone che Le sono vicine se dovesse risultare positiva/o al test genetico?  Are you worried that family and friends will treat you differently if your test result is positive? | \| Per niente  Not at all \| \| Mediamente  Somewhat \| \| \| Molto  Very \| \| \| --- \| --- \| --- \| --- \| --- \| --- \| --- \| \| ❑^1^ \| ❑^2^ \| ❑^3^ \| ❑^4^ \| ❑^5^ \| ❑^6^ \| ❑^7^ \| |

*Vorrebbe parlare di questi argomenti con uno psicologo, oltre che con il genetista? No* ❑^0^ *Sì* ❑^1^

**Would you like to talk to a psychologist in addition to the clinical geneticist about these issues?** *No* ❑^0^ *Yes* ❑^1^

**Figli**

**Children**

| 14. Ha figli?  Do you have any children? | \| ❑^1^ \| ❑^2^ \| Se ha risposto NO passi direttamente alla domanda n. 19  If your answer is NO, skip to question number 19 \| \| --- \| --- \| --- \| \| No  No \| Sì  Yes \| | |  |
| --- | --- | --- | --- | --- | --- | --- | --- | --- |
| 15 a. Si sente in colpa al pensiero di poter aver trasmesso ai suoi figli la Sua eventuale mutazione genetica?  Do you feel guilty about the chance of passing on to your children your possible genetic alterations? | \| ❑^1^ \| ❑^2^ \| Se ha risposto NO passi direttamente alla domanda n. 16  If your answer is NO, skip to question number 16 \| \| --- \| --- \| --- \| \| No  No \| Sì  Yes \| | |  |
| 15b. Quanto si sente in colpa al pensiero di poter aver trasmesso ai Suoi figli la Sua eventuale mutazione genetica? How guilty does the idea of having passed on a genetic mutation to your child/children make you feel? | | \| Per niente  Not at all \| \| Mediamente  Somewhat \| \| \| Molto  Very \| \| \| --- \| --- \| --- \| --- \| --- \| --- \| --- \| \| ❑^1^ \| ❑^2^ \| ❑^3^ \| ❑^4^ \| ❑^5^ \| ❑^6^ \| ❑^7^ \| |  |
| 16. Quanto La preoccupa la reazione che i Suoi figli potrebbero avere nel conoscere il risultato del Suo eventuale test genetico?  Are you worried about how your child/children may react to learning about the results of your genetic test? | | \| Per niente  Not at all \| \| Mediamente  Somewhat \| \| \| Molto  Very \| \| \| --- \| --- \| --- \| --- \| --- \| --- \| --- \| \| ❑^1^ \| ❑^2^ \| ❑^3^ \| ❑^4^ \| ❑^5^ \| ❑^6^ \| ❑^7^ \| |  |
| 17. Quanto La preoccupa la possibilità che il risultato del Suo test abbia ripercussioni sulla relazione con i Suoi figli?  Are you worried that the results of your genetic test may negatively impact on your relationship with your child/children? | | \| Per niente  Not at all \| \| Mediamente  Somewhat \| \| \| Molto  Very \| \| \| --- \| --- \| --- \| --- \| --- \| --- \| --- \| \| ❑^1^ \| ❑^2^ \| ❑^3^ \| ❑^4^ \| ❑^5^ \| ❑^6^ \| ❑^7^ \| |  |
| 18. Le capita spesso di pensare con preoccupazione alla possibilità che i Suoi figli sviluppino un tumore?  Are you worried about the chance of your children developing cancer? | | \| Per niente  Not at all \| \| Mediamente  Somewhat \| \| \| Molto  Very \| \| \| --- \| --- \| --- \| --- \| --- \| --- \| --- \| \| ❑^1^ \| ❑^2^ \| ❑^3^ \| ❑^4^ \| ❑^5^ \| ❑^6^ \| ❑^7^ \| |  |
| *Vorrebbe parlare di questi argomenti con uno psicologo, oltre che con il genetista? No* ❑^0^ *Sì* ❑^1^ | | | |

**Would you like to talk to a psychologist in addition to the clinical geneticist about these issues?** *No* ❑^0^ *Yes* ❑^1^

**Sostegno percepito**

**Perceived social support**

| 19a.Per quel che riguarda la decisione di sottoporsi o meno al test genetico, ne ha parlato con il/la Suo/a compagno/a o coniuge?  About your decision to have genetic testing or not, have you talked about it with your partner? | \| ❑^0^ \| ❑^1^ \| ❑^2^ \| Se non ha un compagno o ha risposto NO passi direttamente alla domanda n. 20  If you do not t have a partner or if you answered NO, skip to question number 20 \| \| --- \| --- \| --- \| --- \| \| Non ho un compagno/coniuge  I do not have a partner \| No  No \| Sì  Yes \| | |  |
| --- | --- | --- | --- | --- | --- | --- | --- | --- | --- | --- |
| 19b. Quanto sostegno sente di avere da parte del/la Suo/a compagno/a o coniuge?  How much support do you feel you have from your partner? | | \| Per niente  Not at all \| \| Mediamente  Somewhat \| \| \| Molto  Very \| \| \| --- \| --- \| --- \| --- \| --- \| --- \| --- \| \| ❑^1^ \| ❑^2^ \| ❑^3^ \| ❑^4^ \| ❑^5^ \| ❑^6^ \| ❑^7^ \| |  |
| 20a.Per quel che riguarda la decisione di sottoporsi o meno al test genetico, ne ha parlato con i Suoi famigliari?  About your decision to have genetic testing or not, have you talked about it with your partner? | \| ❑^1^ \| ❑^2^ \| Se ha risposto NO passi direttamente alla domanda n. 21  If your answer is NO, skip to question number 21 \| \| --- \| --- \| --- \| \| No  No \| Sì  Yes \| | |  |
| 20b. Quanto sostegno sente di avere da parte dei Suoi famigliari?  How much support do you feel you have from your family? | | \| Per niente  Not at all \| \| Mediamente  Somewhat \| \| \| Molto  Very \| \| \| --- \| --- \| --- \| --- \| --- \| --- \| --- \| \| ❑^1^ \| ❑^2^ \| ❑^3^ \| ❑^4^ \| ❑^5^ \| ❑^6^ \| ❑^7^ \| |  |
| 21a.Per quel che riguarda la decisione di sottoporsi o meno al test genetico, ne ha parlato con i Suoi amici?  About your decision to have genetic testing or not, have you talked about it with your friends? | \| ❑^1^ \| ❑^2^ \| Se ha risposto NO passi direttamente alla domanda n. 22  If your answer is NO, skip to question number 22 \| \| --- \| --- \| --- \| \| No  No \| Sì  Yes \| | |  |
| 21b. Quanto sostegno sente di avere da parte dei Suoi amici?  How much support do you feel you have from your friends? | | \| Per niente  Not at all \| \| Mediamente  Somewhat \| \| \| Molto  Very \| \| \| --- \| --- \| --- \| --- \| --- \| --- \| --- \| \| ❑^1^ \| ❑^2^ \| ❑^3^ \| ❑^4^ \| ❑^5^ \| ❑^6^ \| ❑^7^ \| |  |
| *Vorrebbe parlare di questi argomenti con uno psicologo, oltre che con il genetista? No* ❑^0^ *Sì* ❑^1^ | | | |

**Would you like to talk to a psychologist in addition to the clinical geneticist about these issues?** *No* ❑^0^ *Yes* ❑^1^

**Emozioni legate al test genetico**

**Emotions with respect to genetic counseling and testing**

Quando pensa al test genetico, quanto si sente:

When you think about genetic testing, how much do you feel:

| 22. Tesa/o  Tense | \| Per niente  Not at all \| \| Mediamente  Somewhat \| \| \| Molto  Very \| \| \| --- \| --- \| --- \| --- \| --- \| --- \| --- \| \| ❑^1^ \| ❑^2^ \| ❑^3^ \| ❑^4^ \| ❑^5^ \| ❑^6^ \| ❑^7^ \| |  |
| --- | --- | --- | --- | --- | --- | --- | --- | --- | --- | --- | --- | --- | --- | --- | --- | --- |
| 23. Triste  Sad | \| Per niente  Not at all \| \| Mediamente  Somewhat \| \| \| Molto  Very \| \| \| --- \| --- \| --- \| --- \| --- \| --- \| --- \| \| ❑^1^ \| ❑^2^ \| ❑^3^ \| ❑^4^ \| ❑^5^ \| ❑^6^ \| ❑^7^ \| |  |
| 24. Spaventata/o  Scared | \| Per niente  Not at all \| \| Mediamente  Somewhat \| \| \| Molto  Very \| \| \| --- \| --- \| --- \| --- \| --- \| --- \| --- \| \| ❑^1^ \| ❑^2^ \| ❑^3^ \| ❑^4^ \| ❑^5^ \| ❑^6^ \| ❑^7^ \| |  |
| 25. Insicura/o rispetto al futuro  Insecure about the future | \| Per niente  Not at all \| \| Mediamente  Somewhat \| \| \| Molto  Very \| \| \| --- \| --- \| --- \| --- \| --- \| --- \| --- \| \| ❑^1^ \| ❑^2^ \| ❑^3^ \| ❑^4^ \| ❑^5^ \| ❑^6^ \| ❑^7^ \| |  |
| 26. Ottimista  Optimistic | \| Per niente  Not at all \| \| Mediamente  Somewhat \| \| \| Molto  Very \| \| \| --- \| --- \| --- \| --- \| --- \| --- \| --- \| \| ❑^1^ \| ❑^2^ \| ❑^3^ \| ❑^4^ \| ❑^5^ \| ❑^6^ \| ❑^7^ \| |  |
| 27. Serena/o  Calm | \| Per niente  Not at all \| \| Mediamente  Somewhat \| \| \| Molto  Very \| \| \| --- \| --- \| --- \| --- \| --- \| --- \| --- \| \| ❑^1^ \| ❑^2^ \| ❑^3^ \| ❑^4^ \| ❑^5^ \| ❑^6^ \| ❑^7^ \| |  |
| 28. Fiduciosa/o  Confident | \| Per niente  Not at all \| \| Mediamente  Somewhat \| \| \| Molto  Very \| \| \| --- \| --- \| --- \| --- \| --- \| --- \| --- \| \| ❑^1^ \| ❑^2^ \| ❑^3^ \| ❑^4^ \| ❑^5^ \| ❑^6^ \| ❑^7^ \| |  |
| 29. In grado di gestire la situazione  Able to handle the situation | \| Per niente  Not at all \| \| Mediamente  Somewhat \| \| \| Molto  Very \| \| \| --- \| --- \| --- \| --- \| --- \| --- \| --- \| \| ❑^1^ \| ❑^2^ \| ❑^3^ \| ❑^4^ \| ❑^5^ \| ❑^6^ \| ❑^7^ \| |  |
| *Vorrebbe parlare di questi argomenti con uno psicologo, oltre che con il genetista? No* ❑^0^ *Sì* ❑^1^ | | |
|  |  |  |

**Would you like to talk to a psychologist in addition to the clinical geneticist about these issues?** *No* ❑^0^ *Yes* ❑^1^

**L’esperienza della malattia**

**Living with cancer**

| 30. Se attualmente ha un tumore, quanto è emotivamente faticoso per Lei affrontarlo?  If you have cancer now, how emotionally burdensome is it for you? | | Non ho un tumore  I do not have cancer now  ❑^0^ | \| Per niente  Not at all \| \| Mediamente  Somewhat \| \| \| Molto  Very \| \| \| --- \| --- \| --- \| --- \| --- \| --- \| --- \| \| ❑^1^ \| ❑^2^ \| ❑^3^ \| ❑^4^ \| ❑^5^ \| ❑^6^ \| ❑^7^ \| |
| --- | --- | --- | --- | --- | --- | --- | --- | --- | --- | --- | --- | --- | --- | --- | --- | --- | --- |
|  |  |  |  |
| 31. Se ha avuto un tumore precedente, quanto è stato emotivamente faticoso per Lei affrontarlo?  If you had cancer in the past, how emotionally burdensome was it for you? | | Non ho avuto un tumore  I have not had cancer in the past  ❑^0^ | \| Per niente  Not at all \| \| Mediamente  Somewhat \| \| \| Molto  Very \| \| \| --- \| --- \| --- \| --- \| --- \| --- \| --- \| \| ❑^1^ \| ❑^2^ \| ❑^3^ \| ❑^4^ \| ❑^5^ \| ❑^6^ \| ❑^7^ \| |
|  |  |  |  |
| 32. Le capita spesso di pensare con preoccupazione alla possibilità di sviluppare un tumore o un nuovo tumore?  Are you worried about the chance of getting cancer (again)? | | | \| Per niente  Not at all \| \| Mediamente  Somewhat \| \| \| Molto  Very \| \| \| --- \| --- \| --- \| --- \| --- \| --- \| --- \| \| ❑^1^ \| ❑^2^ \| ❑^3^ \| ❑^4^ \| ❑^5^ \| ❑^6^ \| ❑^7^ \| |
| 33. Se ha avuto famigliari malati di tumore, quanto impatto ha avuto questo sulla Sua vita?  If you have had family members with cancer, how did it impact on your life? | Non ho avuto famigliari con tumore  I have not had any family members with cancer  ❑^0^ | | \| Per niente  Not at all \| \| Mediamente  Somewhat \| \| \| Molto  Very \| \| \| --- \| --- \| --- \| --- \| --- \| --- \| --- \| \| ❑^1^ \| ❑^2^ \| ❑^3^ \| ❑^4^ \| ❑^5^ \| ❑^6^ \| ❑^7^ \| |
|  |  | |  |

| *Vorrebbe parlare di questi argomenti con uno psicologo, oltre che con il genetista? No* ❑^0^ *Sì* ❑^1^ |
| --- |
|  |

**Would you like to talk to a psychologist in addition to the clinical geneticist about these issues?** *No* ❑^0^ *Yes* ❑^1^

# **SM 1 - Description of the procedure used in the pilot study on item readability**

The individuals who were scheduled for an appointment in the following days were contacted by telephone to invite them to participate in the study. Those who accepted were e-mailed with the information of the study and the informed consent. On the day of the interview the person was first asked to complete the questionnaire and afterwards underwent a semi-structured audio-recorded interview with a licensed psychologist. During the interview the psychologist investigated whether each item was difficult to answer, confusing, difficult to understand, shocking, offensive, and if its understandability could be improved if some words were changed. Interviewees were also asked to rephrase each item in their own words to test whether the meaning of the item was understood as intended by the developers. It was also investigated whether the instructions were clear, whether the answer scale was easy to use, whether there were repeated or redundant questions, whether the graphical layout and the length of the questionnaire were adequate, and whether the questionnaire was perceived as useful. At the end of the interview further comments about the item content were encouraged. Finally, the psychologist made sure that the interviewee did not show any unpleasant feelings, thanked her/him, and dismissed her/him.

# **SM 2 - Descriptive statistics of the I-PAHC items**

### Table SM2.1 Descriptive statistics for the item I-PACH1 How much are you motivated to having the test?

| Centre | Valid | Missing | Mean | SD | Min | Max | Q1 | Median | Q3 |
| --- | --- | --- | --- | --- | --- | --- | --- | --- | --- |
| Total | 265 | 6 | 6.15 | 1.28 | 1 | 7 | 5 | 7 | 7 |
| Bologna | 56 | 1 | 6.50 | 1.01 | 3 | 7 | 7 | 7 | 7 |
| Genova | 152 | 2 | 6.11 | 1.29 | 1 | 7 | 5 | 7 | 7 |
| Lecce | 37 | 3 | 6.08 | 1.48 | 2 | 7 | 6 | 7 | 7 |
| Modena | 20 | 0 | 5.65 | 1.31 | 3 | 7 | 4 | 6 | 7 |

*Note*: SD: standard deviation; Min: minimum value; Max: maximum value; Q1 and Q3: first and third quartile, respectively.

### Table SM2.2 Descriptive statistics for the item I-PACH2 Have you discussed your decision to have the test or not with your family doctor or a specialist physician?

| Centre | No | Yes, useful | Yes, not useful | Total |
| --- | --- | --- | --- | --- |
| Bologna | NA | NA | NA | NA |
|  | NA | NA | NA | NA |
| Genova | 63 | 77 | 10 | 150 |
|  | 42.00% | 51.33% | 6.67% |  |
| Lecce | 4 | 33 | 0 | 37 |
|  | 10.81% | 89.19% | 0.00% |  |
| Modena | 5 | 13 | 0 | 18 |
|  | 27.78% | 72.22% | 0.00% |  |
| Total | 72 | 123 | 10 | 205 |
|  | 35.12% | 60.00% | 4.88% |  |

### Table SM2.2a Descriptive statistics for the item I-PACH2a - Please report any healthcare professional you consulted:

|  | Centre | | | |  |
| --- | --- | --- | --- | --- | --- |
| Specialist | Bologna | Genova | Lecce | Modena | Total |
| Geneticist | 31.5% | 3.3% | 19.4% | 0.0% | 11.0% |
| General Practitioner | 23.6% | 15.0% | 29.7% | 15.0% | 18.9% |
| Gynaecologist | 10.9% | 15.7% | 27.0% | 19.0% | 15.8% |
| Oncologist | 34.5% | 42.5% | 51.4% | 65.0% | 44.5% |
| Nurse | 3.6% | 0.7% | 0.0% | 0.0% | 1.1% |
| Surgeon | 10.9% | 6.6% | 8.1% | 10.0% | 8.0% |
| Psychologist | 3.6% | 5.2% | 2.7% | 20.0% | 5.7% |
| Other | 9.3% | 3.3% | 5.4% | 0.0% | 4.6% |

### Table SM2.3 Descriptive statistics for the item I-PACH3 - Are you worried about the chance of being a carrier of a genetic mutation?

| Centre | Valid | Missing | Mean | SD | Min | Max | Q1 | Median | Q3 |
| --- | --- | --- | --- | --- | --- | --- | --- | --- | --- |
| Total | 266 | 5 | 5.12 | 1.71 | 1 | 7 | 4 | 5 | 7 |
| Bologna | 56 | 1 | 5.13 | 1.79 | 1 | 7 | 4 | 5 | 7 |
| Genova | 153 | 1 | 4.98 | 1.71 | 1 | 7 | 4 | 5 | 7 |
| Lecce | 37 | 3 | 5.68 | 1.56 | 1 | 7 | 4 | 6 | 7 |
| Modena | 20 | 0 | 5.15 | 1.63 | 1 | 7 | 4 | 5 | 7 |

*Note*: SD: standard deviation; Min: minimum value; Max: maximum value; Q1 and Q3: first and third quartile, respectively.

### Table SM2.4 Descriptive statistics for the item I-PACH4 - Are you worried about the impact the genetic test may have on your life?

| Centre | Valid | Missing | Mean | SD | Min | Max | Q1 | Median | Q3 |
| --- | --- | --- | --- | --- | --- | --- | --- | --- | --- |
| Total | 265 | 6 | 4.38 | 1.86 | 1 | 7 | 3 | 4 | 6 |
| Bologna | 55 | 2 | 4.40 | 1.98 | 1 | 7 | 3 | 4 | 6 |
| Genova | 153 | 1 | 4.16 | 1.81 | 1 | 7 | 3 | 4 | 5 |
| Lecce | 37 | 3 | 5.30 | 1.58 | 1 | 7 | 4 | 5 | 7 |
| Modena | 20 | 0 | 4.25 | 2.00 | 1 | 7 | 3 | 4 | 6 |

*Note*: SD: standard deviation; Min: minimum value; Max: maximum value; Q1 and Q3: first and third quartile, respectively.

### Table SM2.5 Descriptive statistics for the item I-PACH5 - Are you worried about having to have intensified screening (more frequent appointments)?

| Centre | Valid | Missing | Mean | SD | Min | Max | Q1 | Median | Q3 |
| --- | --- | --- | --- | --- | --- | --- | --- | --- | --- |
| Total | 266 | 5 | 3.87 | 1.78 | 1 | 7 | 2 | 4 | 5 |
| Bologna | 56 | 1 | 3.71 | 1.80 | 1 | 7 | 2 | 4 | 5 |
| Genova | 153 | 1 | 3.85 | 1.81 | 1 | 7 | 2 | 4 | 5 |
| Lecce | 37 | 3 | 4.41 | 1.61 | 1 | 7 | 4 | 5 | 5 |
| Modena | 20 | 0 | 3.45 | 1.67 | 1 | 7 | 2 | 3 | 4 |

*Note*: SD: standard deviation; Min: minimum value; Max: maximum value; Q1 and Q3: first and third quartile, respectively.

### Table SM2.6 Descriptive statistics for the item I-PACH6 - Are you worried about having to have cancer risk reducing surgery?

| Centre | Valid | Missing | Mean | SD | Min | Max | Q1 | Median | Q3 |
| --- | --- | --- | --- | --- | --- | --- | --- | --- | --- |
| Total | 263 | 8 | 4.90 | 1.84 | 1 | 7 | 4 | 5 | 6 |
| Bologna | 54 | 3 | 4.93 | 2.02 | 1 | 7 | 4 | 5 | 7 |
| Genova | 153 | 1 | 4.81 | 1.82 | 1 | 7 | 4 | 5 | 6 |
| Lecce | 36 | 4 | 5.22 | 1.74 | 1 | 7 | 4 | 6 | 6 |
| Modena | 20 | 0 | 5.00 | 1.62 | 1 | 7 | 4 | 5 | 6 |

*Note*: SD: standard deviation; Min: minimum value; Max: maximum value; Q1 and Q3: first and third quartile, respectively.

### Table SM2.7 Descriptive statistics for the item I-PACH7 - Are you worried about the impact that hereditary susceptibility to cancer could have on your life plans?

| Centre | Valid | Missing | Mean | SD | Min | Max | Q1 | Median | Q3 |
| --- | --- | --- | --- | --- | --- | --- | --- | --- | --- |
| Total | 265 | 6 | 4.54 | 1.76 | 1 | 7 | 3 | 5 | 6 |
| Bologna | 56 | 1 | 4.71 | 1.65 | 1 | 7 | 3 | 5 | 6 |
| Genova | 152 | 2 | 4.43 | 1.75 | 1 | 7 | 3 | 5 | 6 |
| Lecce | 37 | 3 | 5.14 | 1.81 | 1 | 7 | 4 | 6 | 6 |
| Modena | 20 | 0 | 3.80 | 1.74 | 1 | 7 | 3 | 3 | 5 |

*Note*: SD: standard deviation; Min: minimum value; Max: maximum value; Q1 and Q3: first and third quartile, respectively.

### Table SM2.8 Descriptive statistics for the item I-PACH8 - Are you worried about the fact that doctors still do not fully understand hereditary susceptibility to cancer (e.g., what certain mutations mean or how best to manage hereditary cancer risk)?

| Centre | Valid | Missing | Mean | SD | Min | Max | Q1 | Median | Q3 |
| --- | --- | --- | --- | --- | --- | --- | --- | --- | --- |
| Total | 265 | 6 | 4.97 | 1.84 | 1 | 7 | 4 | 5 | 7 |
| Bologna | 55 | 2 | 5.15 | 1.66 | 1 | 7 | 4 | 5 | 7 |
| Genova | 153 | 1 | 5.13 | 1.72 | 1 | 7 | 4 | 5 | 7 |
| Lecce | 37 | 3 | 5.35 | 1.74 | 1 | 7 | 4 | 6 | 7 |
| Modena | 20 | 0 | 2.55 | 1.70 | 1 | 7 | 2 | 2 | 3 |

*Note*: SD: standard deviation; Min: minimum value; Max: maximum value; Q1 and Q3: first and third quartile, respectively.

### Table SM2.8PSY1 Crosstab Centre × answer to the question "Would you like to talk to a psychologist in addition to the clinical geneticist about these issues?" in the section "Hereditary predisposition" (counts and row percentages)

| Centre | No | Yes | Total |
| --- | --- | --- | --- |
| Bologna | 41 | 12 | 53 |
|  | 77.36% | 22.64% |  |
| Genova | 78 | 59 | 137 |
|  | 56.93% | 43.07% |  |
| Lecce | 21 | 14 | 35 |
|  | 60.00% | 40.00% |  |
| Modena | 12 | 8 | 20 |
|  | 60.00% | 40.00% |  |
| Total | 152 | 93 | 245 |
|  | 62.04% | 37.96% |  |

### Table SM2.9 Descriptive statistics for the item I-PACH9 - Are you worried about sharing the results of your genetic test result with your family?

| Centre | Valid | Missing | Mean | SD | Min | Max | Q1 | Median | Q3 |
| --- | --- | --- | --- | --- | --- | --- | --- | --- | --- |
| Total | 266 | 5 | 3.81 | 2.17 | 1 | 7 | 2 | 4 | 6 |
| Bologna | 57 | 0 | 3.40 | 2.23 | 1 | 7 | 1 | 3 | 5 |
| Genova | 152 | 2 | 3.81 | 2.19 | 1 | 7 | 2 | 4 | 6 |
| Lecce | 37 | 3 | 4.65 | 2.02 | 1 | 7 | 3 | 5 | 7 |
| Modena | 20 | 0 | 3.45 | 1.70 | 1 | 6 | 2 | 4 | 4 |

*Note*: SD: standard deviation; Min: minimum value; Max: maximum value; Q1 and Q3: first and third quartile, respectively.

### Table SM2.10 Descriptive statistics for the item I-PACH10 - Are you worried about informing your relatives that they could benefit from having the genetic test?

| Centre | Valid | Missing | Mean | SD | Min | Max | Q1 | Median | Q3 |
| --- | --- | --- | --- | --- | --- | --- | --- | --- | --- |
| Total | 266 | 5 | 3.47 | 2.17 | 1 | 7 | 1 | 3 | 5 |
| Bologna | 57 | 0 | 3.40 | 2.24 | 1 | 7 | 1 | 3 | 6 |
| Genova | 152 | 2 | 3.34 | 2.20 | 1 | 7 | 1 | 3 | 5 |
| Lecce | 37 | 3 | 4.24 | 2.02 | 1 | 7 | 2 | 5 | 6 |
| Modena | 20 | 0 | 3.20 | 1.88 | 1 | 6 | 1 | 4 | 5 |

*Note*: SD: standard deviation; Min: minimum value; Max: maximum value; Q1 and Q3: first and third quartile, respectively.

### Table SM2.11 Descriptive statistics for the item I-PACH11 - Are you worried about the chance that a family member gets cancer?

| Centre | Valid | Missing | Mean | SD | Min | Max | Q1 | Median | Q3 |
| --- | --- | --- | --- | --- | --- | --- | --- | --- | --- |
| Total | 267 | 4 | 5.58 | 1.69 | 1 | 7 | 4 | 6 | 7 |
| Bologna | 57 | 0 | 5.89 | 1.70 | 1 | 7 | 5 | 7 | 7 |
| Genova | 153 | 1 | 5.40 | 1.71 | 1 | 7 | 4 | 6 | 7 |
| Lecce | 37 | 3 | 6.19 | 1.31 | 2 | 7 | 6 | 7 | 7 |
| Modena | 20 | 0 | 4.90 | 1.68 | 2 | 7 | 4 | 5 | 6 |

*Note*: SD: standard deviation; Min: minimum value; Max: maximum value; Q1 and Q3: first and third quartile, respectively.

### Table SM2.12 Descriptive statistics for the item I-PACH12 - About your decision to have the test or not, how much do you feel the responsibility towards your family members?

| Centre | Valid | Missing | Mean | SD | Min | Max | Q1 | Median | Q3 |
| --- | --- | --- | --- | --- | --- | --- | --- | --- | --- |
| Total | 265 | 6 | 4.17 | 2.21 | 1 | 7 | 2 | 4 | 6 |
| Bologna | 55 | 2 | 4.16 | 2.35 | 1 | 7 | 2 | 4 | 7 |
| Genova | 153 | 1 | 3.98 | 2.22 | 1 | 7 | 2 | 4 | 6 |
| Lecce | 37 | 3 | 5.43 | 1.50 | 2 | 7 | 5 | 6 | 7 |
| Modena | 20 | 0 | 3.25 | 2.02 | 1 | 7 | 2 | 3 | 4 |

*Note*: SD: standard deviation; Min: minimum value; Max: maximum value; Q1 and Q3: first and third quartile, respectively.

### Table SM2.13 Descriptive statistics for the item I-PACH13 - Are you worried that family and friends will treat you differently if your test result is positive?

| Centre | Valid | Missing | Mean | SD | Min | Max | Q1 | Median | Q3 |
| --- | --- | --- | --- | --- | --- | --- | --- | --- | --- |
| Total | 264 | 7 | 2.61 | 1.87 | 1 | 7 | 1 | 2 | 4 |
| Bologna | 54 | 3 | 2.46 | 1.93 | 1 | 7 | 1 | 2 | 4 |
| Genova | 153 | 1 | 2.55 | 1.78 | 1 | 7 | 1 | 2 | 3 |
| Lecce | 37 | 3 | 3.19 | 2.13 | 1 | 7 | 1 | 3 | 5 |
| Modena | 20 | 0 | 2.35 | 1.76 | 1 | 7 | 1 | 2 | 3 |

*Note*: SD: standard deviation; Min: minimum value; Max: maximum value; Q1 and Q3: first and third quartile, respectively.

### Table SM2.13PSY2 Crosstab Centre × answer to the question "Would you like to talk to a psychologist in addition to the clinical geneticist about these issues?" in the section "Family and social environment" (counts and row percentages)

| Centre | No | Yes | Total |
| --- | --- | --- | --- |
| Bologna | 37 | 13 | 50 |
|  | 74.00% | 26.00% |  |
| Genova | 77 | 52 | 129 |
|  | 59.69% | 40.31% |  |
| Lecce | 21 | 15 | 36 |
|  | 58.33% | 41.67% |  |
| Modena | 11 | 9 | 20 |
|  | 55.00% | 45.00% |  |
| Total | 146 | 89 | 235 |
|  | 62.13% | 37.87% |  |

### Table SM2.14 Crosstab Centre × answer to the question I-PACH14 Do you have children? (counts and row percentages)

| Centre | No | Yes | Total |
| --- | --- | --- | --- |
| Bologna | 9 | 47 | 56 |
|  | 16.07% | 83.93% |  |
| Genova | 41 | 112 | 153 |
|  | 26.80% | 73.20% |  |
| Lecce | 8 | 29 | 37 |
|  | 21.62% | 78.38% |  |
| Modena | 2 | 18 | 20 |
|  | 10.00% | 90.00% |  |
| Total | 60 | 206 | 266 |
|  | 22.56% | 77.44% |  |

### Table SM2.15a Crosstab Centre × answer to the question I-PACH15a Do you feel guilty about the chance of passing on to your children your possible genetic alterations? (counts and row percentages)

| Centre | No | Yes | Total |
| --- | --- | --- | --- |
| Bologna | 18 | 27 | 45 |
|  | 40.00% | 60.00% |  |
| Genova | 59 | 52 | 111 |
|  | 53.15% | 46.85% |  |
| Lecce | 9 | 20 | 29 |
|  | 31.03% | 68.97% |  |
| Modena | 5 | 13 | 18 |
|  | 27.78% | 72.22% |  |
| Total | 91 | 112 | 203 |
|  | 44.83% | 55.17% |  |

### Table SM2.15b Descriptive statistics for the item I-PACH15b - How guilty does the idea of having passed on a genetic mutation to your child/children make you feel?

| Centre | Valid | Missing | Mean | SD | Min | Max | Q1 | Median | Q3 |
| --- | --- | --- | --- | --- | --- | --- | --- | --- | --- |
| Total | 155 | 116 | 5.03 | 2.10 | 1 | 7 | 4 | 6 | 7 |
| Bologna | 38 | 19 | 5.11 | 2.33 | 1 | 7 | 3 | 6 | 7 |
| Genova | 79 | 75 | 4.73 | 2.16 | 1 | 7 | 3 | 5 | 7 |
| Lecce | 25 | 15 | 5.44 | 1.80 | 1 | 7 | 4 | 6 | 7 |
| Modena | 13 | 7 | 5.77 | 1.24 | 4 | 7 | 5 | 6 | 7 |

*Note*: SD: standard deviation; Min: minimum value; Max: maximum value; Q1 and Q3: first and third quartile, respectively.

### Table SM2.16 Descriptive statistics for the item I-PACH16 - Are you worried about how your child/children may react to learning about the results of your genetic test?

| Centre | Valid | Missing | Mean | SD | Min | Max | Q1 | Median | Q3 |
| --- | --- | --- | --- | --- | --- | --- | --- | --- | --- |
| Total | 203 | 68 | 4.33 | 2.05 | 1 | 7 | 3 | 4 | 6 |
| Bologna | 47 | 10 | 3.91 | 2.15 | 1 | 7 | 2 | 4 | 6 |
| Genova | 109 | 45 | 4.36 | 2.03 | 1 | 7 | 3 | 5 | 6 |
| Lecce | 29 | 11 | 4.52 | 2.05 | 1 | 7 | 3 | 5 | 6 |
| Modena | 18 | 2 | 4.89 | 1.88 | 1 | 7 | 4 | 5 | 6 |

*Note*: SD: standard deviation; Min: minimum value; Max: maximum value; Q1 and Q3: first and third quartile, respectively.

### Table SM2.17 Descriptive statistics for the item I-PACH17 - Are you worried that the results of your genetic test may negatively impact on your relationship with your child/children?

| Centre | Valid | Missing | Mean | SD | Min | Max | Q1 | Median | Q3 |
| --- | --- | --- | --- | --- | --- | --- | --- | --- | --- |
| Total | 204 | 67 | 3.25 | 2.19 | 1 | 7 | 1 | 3 | 5 |
| Bologna | 47 | 10 | 3.70 | 2.28 | 1 | 7 | 1 | 3 | 6 |
| Genova | 110 | 44 | 3.16 | 2.14 | 1 | 7 | 1 | 2 | 5 |
| Lecce | 29 | 11 | 3.24 | 2.21 | 1 | 7 | 1 | 3 | 5 |
| Modena | 18 | 2 | 2.56 | 2.06 | 1 | 7 | 1 | 2 | 3 |

*Note*: SD: standard deviation; Min: minimum value; Max: maximum value; Q1 and Q3: first and third quartile, respectively.

### Table SM2.18 Descriptive statistics for the item I-PACH18 - Are you worried about the chance of your children developing cancer?

| Centre | Valid | Missing | Mean | SD | Min | Max | Q1 | Median | Q3 |
| --- | --- | --- | --- | --- | --- | --- | --- | --- | --- |
| Total | 203 | 68 | 5.22 | 1.96 | 1 | 7 | 4 | 6 | 7 |
| Bologna | 46 | 11 | 5.30 | 2.07 | 1 | 7 | 3 | 7 | 7 |
| Genova | 110 | 44 | 4.94 | 1.95 | 1 | 7 | 3 | 5 | 7 |
| Lecce | 29 | 11 | 5.90 | 1.80 | 1 | 7 | 6 | 7 | 7 |
| Modena | 18 | 2 | 5.67 | 1.68 | 1 | 7 | 5 | 6 | 7 |

*Note*: SD: standard deviation; Min: minimum value; Max: maximum value; Q1 and Q3: first and third quartile, respectively.

### Table SM2.18PSY3 Crosstab Centre × answer to the question "Would you like to talk to a psychologist in addition to the clinical geneticist about these issues?" in the section "Children" (counts and row percentages)

| Centre | No | Yes | Total |
| --- | --- | --- | --- |
| Bologna | 31 | 12 | 43 |
|  | 72.09% | 27.91% |  |
| Genova | 66 | 37 | 103 |
|  | 64.08% | 35.92% |  |
| Lecce | 16 | 12 | 28 |
|  | 57.14% | 42.86% |  |
| Modena | 5 | 13 | 18 |
|  | 27.78% | 72.22% |  |
| Total | 118 | 74 | 192 |
|  | 61.46% | 38.54% |  |

*Note*: SD: standard deviation; Min: minimum value; Max: maximum value; Q1 and Q3: first and third quartile, respectively.

### Table SM2.19a Crosstab Centre × Having a partner (I-PACH19a) (counts and row percentages)

| Centre | No | Yes | Total |
| --- | --- | --- | --- |
| Bologna | 10 | 45 | 55 |
|  | 18.18% | 81.82% |  |
| Genova | 33 | 116 | 149 |
|  | 22.15% | 77.85% |  |
| Lecce | 1 | 36 | 37 |
|  | 2.70% | 97.30% |  |
| Modena | 4 | 16 | 20 |
|  | 20.00% | 80.00% |  |
| Total | 48 | 213 | 261 |
|  | 18.39% | 81.61% |  |

### Table SM2.19a-bis Crosstab Centre × answer to the question I-PACH19a - About your decision to have genetic testing or not, have you talked about it with your partner (counts and row percentages)

| Centre | No | Yes | Total |
| --- | --- | --- | --- |
| Bologna | 9 | 36 | 45 |
|  | 20.00% | 80.00% |  |
| Genova | 7 | 109 | 116 |
|  | 6.03% | 93.97% |  |
| Lecce | 1 | 35 | 36 |
|  | 2.78% | 97.22% |  |
| Modena | 8 | 8 | 16 |
|  | 50.00% | 50.00% |  |
| Total | 25 | 188 | 213 |
|  | 11.74% | 88.26% |  |

### Table SM2.19b Descriptive statistics for the item I-PACH19b - How much support do you feel you have from your partner?

| Centre | Valid | Missing | Mean | SD | Min | Max | Q1 | Median | Q3 |
| --- | --- | --- | --- | --- | --- | --- | --- | --- | --- |
| Total | 198 | 15 | 6.04 | 1.61 | 1 | 7 | 5 | 7 | 7 |
| Bologna | 40 | 5 | 6.18 | 1.48 | 1 | 7 | 5 | 7 | 7 |
| Genova | 111 | 5 | 5.84 | 1.77 | 1 | 7 | 5 | 7 | 7 |
| Lecce | 34 | 2 | 6.62 | 1.07 | 2 | 7 | 7 | 7 | 7 |
| Modena | 13 | 3 | 5.77 | 1.54 | 2 | 7 | 5 | 6 | 7 |

*Note*: SD: standard deviation; Min: minimum value; Max: maximum value; Q1 and Q3: first and third quartile, respectively.

### Table SM2.20a Crosstab Centre × answer to the question I-PAHC20a About your decision to have genetic testing or not, have you talked about it with your partner (counts and row percentages)

| Centre | No | Yes | Total |
| --- | --- | --- | --- |
| Bologna | 7 | 45 | 52 |
|  | 13.46% | 86.54% |  |
| Genova | 22 | 121 | 143 |
|  | 15.38% | 84.62% |  |
| Lecce | 3 | 34 | 37 |
|  | 8.11% | 91.89% |  |
| Modena | 10 | 7 | 17 |
|  | 58.82% | 41.18% |  |
| Total | 42 | 207 | 249 |
|  | 16.87% | 83.13% |  |

### Table SM2.20b Descriptive statistics for the item I-PACH20b - How much support do you feel you have from your family?

| Centre | Valid | Missing | Mean | SD | Min | Max | Q1 | Median | Q3 |
| --- | --- | --- | --- | --- | --- | --- | --- | --- | --- |
| Total | 183 | 30 | 6.08 | 1.51 | 1 | 7 | 6 | 7 | 7 |
| Bologna | 41 | 4 | 6.24 | 1.56 | 1 | 7 | 6 | 7 | 7 |
| Genova | 101 | 15 | 5.89 | 1.62 | 1 | 7 | 5 | 7 | 7 |
| Lecce | 32 | 4 | 6.53 | 1.02 | 3 | 7 | 7 | 7 | 7 |
| Modena | 9 | 7 | 5.78 | 1.20 | 4 | 7 | 5 | 5 | 7 |

*Note*: SD: standard deviation; Min: minimum value; Max: maximum value; Q1 and Q3: first and third quartile, respectively.

### Table SM2.21a Crosstab Centre × I-PACH21a - About your decision to have genetic testing or not, have you talked about it with your friends (counts and row percentages)

| Centre | No | Yes | Total |
| --- | --- | --- | --- |
| Bologna | 28 | 27 | 55 |
|  | 50.91% | 49.09% |  |
| Genova | 58 | 92 | 150 |
|  | 38.67% | 61.33% |  |
| Lecce | 23 | 14 | 37 |
|  | 62.16% | 37.84% |  |
| Modena | 16 | 2 | 18 |
|  | 88.89% | 11.11% |  |
| Total | 125 | 135 | 260 |
|  | 48.08% | 51.92% |  |

### Table SM2.21b Descriptive statistics for the item I-PACH 21b - How much support do you feel you have from your friends?

| Centre | Valid | Missing | Mean | SD | Min | Max | Q1 | Median | Q3 |
| --- | --- | --- | --- | --- | --- | --- | --- | --- | --- |
| Total | 169 | 102 | 5.32 | 1.89 | 1 | 7 | 4 | 6 | 7 |
| Bologna | 35 | 22 | 5.43 | 1.84 | 1 | 7 | 4 | 6 | 7 |
| Genova | 109 | 45 | 5.19 | 1.92 | 1 | 7 | 4 | 6 | 7 |
| Lecce | 21 | 19 | 5.52 | 1.91 | 1 | 7 | 5 | 6 | 7 |
| Modena | 4 | 16 | 6.75 | 0.50 | 6 | 7 | 7 | 7 | 7 |

*Note*: SD: standard deviation; Min: minimum value; Max: maximum value; Q1 and Q3: first and third quartile, respectively.

### Table SM2.21PSY4 Crosstab Centre × answer to the question "Would you like to talk to a psychologist in addition to the clinical geneticist about these issues?" in the section "Perceived Social Support" (counts and row percentages)

| Centre | No | Yes | Total |
| --- | --- | --- | --- |
| Bologna | 32 | 11 | 43 |
|  | 74.42% | 25.58% |  |
| Genova | 75 | 31 | 106 |
|  | 70.75% | 29.25% |  |
| Lecce | 21 | 8 | 29 |
|  | 72.41% | 27.59% |  |
| Modena | 14 | 6 | 20 |
|  | 70.00% | 30.00% |  |
| Total | 142 | 56 | 198 |
|  | 71.72% | 28.28% |  |

### Table SM2.21PSY4a Correlations between the items of the section Perceived Social Support and answer to the question "Would you like to talk to a psychologist in addition to the clinical geneticist about these issues?"

| Item | Total^§^ | Bologna | Genova | Lecce | Modena |
| --- | --- | --- | --- | --- | --- |
| pahc19a° | .04 | .13 | .00 | NC | NC |
| pahc19b^ | -.05 | -.10 | .01 | -.18 | .15 |
| pahc20a° | .04 | .07 | .10 | NC | NC |
| pahc20b^ | -.20** | -.47** | -.15 | -.25 | .26 |
| pahc21a° | .18* | .37* | .13 | .15 | NC |
| pahc21b^ | .04 | .03 | .04 | .05 | .33 |

*Note*: §: corrected for the nesting of participants into centres. °: dichotomized variable (No/Yes), *phi* coefficient; ^: variable that takes into account only cases that answered "Yes" to the question and provided quantitative information; *Kendall's tau* coefficient; **: *p* <. 01; *: *p* <. 05; NC: not computable

### Table SM2.22_29 Descriptive statistics for the items of the section "Emotions with respect to to genetic counseling and testing"

| Centre | Item | Valid | Missing | M | SD | min | max | Q1 | Median | Q3 |
| --- | --- | --- | --- | --- | --- | --- | --- | --- | --- | --- |
| Total | pahc22-Tense | 254 | 17 | 3.49 | 1.91 | 1 | 7 | 2 | 3 | 5 |
|  | pahc23-Sad | 251 | 20 | 2.73 | 1.75 | 1 | 7 | 1 | 2 | 4 |
|  | pahc24-Scared | 253 | 18 | 3.47 | 2.04 | 1 | 7 | 2 | 3 | 5 |
|  | pahc25-Insecure about the future | 253 | 18 | 3.79 | 2.03 | 1 | 7 | 2 | 4 | 5 |
|  | pahc26-Optimistic | 255 | 16 | 4.35 | 1.80 | 1 | 7 | 3 | 4 | 6 |
|  | pahc27-Calm | 255 | 16 | 4.07 | 1.67 | 1 | 7 | 3 | 4 | 5 |
|  | pahc28-Confident | 258 | 13 | 4.78 | 1.64 | 1 | 7 | 4 | 5 | 6 |
|  | pahc29- Able to handle the situation | 261 | 10 | 5.01 | 1.39 | 1 | 7 | 4 | 5 | 6 |
|  |  |  |  |  |  |  |  |  |  |  |
| Bologna | pahc22-Tense | 53 | 4 | 3.47 | 2.04 | 1 | 7 | 2 | 4 | 5 |
|  | pahc23-Sad | 53 | 4 | 2.92 | 1.90 | 1 | 7 | 1 | 2 | 4 |
|  | pahc24-Scared | 53 | 4 | 3.64 | 2.18 | 1 | 7 | 2 | 4 | 5 |
|  | pahc25-Insecure about the future | 53 | 4 | 3.81 | 2.13 | 1 | 7 | 2 | 4 | 6 |
|  | pahc26-Optimistic | 55 | 2 | 4.60 | 1.85 | 1 | 7 | 4 | 4 | 7 |
|  | pahc27-Calm | 54 | 3 | 4.31 | 1.54 | 1 | 7 | 3 | 4 | 5 |
|  | pahc28-Confident | 53 | 4 | 4.94 | 1.75 | 1 | 7 | 4 | 5 | 7 |
|  | pahc29- Able to handle the situation | 54 | 3 | 4.98 | 1.43 | 1 | 7 | 4 | 5 | 6 |
|  |  |  |  |  |  |  |  |  |  |  |
| Genova | pahc22-Tense | 145 | 9 | 3.26 | 1.79 | 1 | 7 | 2 | 3 | 5 |
|  | pahc23-Sad | 142 | 12 | 2.46 | 1.61 | 1 | 7 | 1 | 2 | 3 |
|  | pahc24-Scared | 144 | 10 | 3.06 | 1.89 | 1 | 7 | 2 | 3 | 4 |
|  | pahc25-Insecure about the future | 144 | 10 | 3.50 | 1.93 | 1 | 7 | 2 | 3 | 5 |
|  | pahc26-Optimistic | 144 | 10 | 4.39 | 1.84 | 1 | 7 | 3 | 4 | 6 |
|  | pahc27-Calm | 145 | 9 | 4.21 | 1.67 | 1 | 7 | 3 | 4 | 5 |
|  | pahc28-Confident | 149 | 5 | 4.83 | 1.55 | 1 | 7 | 4 | 5 | 6 |
|  | pahc29- Able to handle the situation | 150 | 4 | 5.01 | 1.39 | 2 | 7 | 4 | 5 | 6 |

continues

### Table SM2.22_29 Descriptive statistics for the items of the section "Emotions with respect to to genetic counseling and testing" (continued)

| Centre | Item | Valid | Missing | M | SD | min | max | Q1 | Median | Q3 |
| --- | --- | --- | --- | --- | --- | --- | --- | --- | --- | --- |
| Lecce | pahc22-Tense | 36 | 4 | 4.42 | 1.95 | 1 | 7 | 3 | 5 | 6 |
|  | pahc23-Sad | 36 | 4 | 3.56 | 1.87 | 1 | 7 | 2 | 4 | 5 |
|  | pahc24-Scared | 36 | 4 | 4.50 | 2.02 | 1 | 7 | 3 | 5 | 6 |
|  | pahc25-Insecure about the future | 36 | 4 | 4.36 | 2.23 | 1 | 7 | 3 | 4 | 7 |
|  | pahc26-Optimistic | 36 | 4 | 4.53 | 1.58 | 1 | 7 | 4 | 4 | 5 |
|  | pahc27-Calm | 36 | 4 | 3.78 | 1.77 | 1 | 7 | 2 | 4 | 5 |
|  | pahc28-Confident | 36 | 4 | 4.89 | 1.70 | 1 | 7 | 4 | 5 | 6 |
|  | pahc29- Able to handle the situation | 37 | 3 | 5.05 | 1.54 | 1 | 7 | 4 | 5 | 6 |
|  |  |  |  |  |  |  |  |  |  |  |
| Modena | pahc22-Tense | 20 | 0 | 3.55 | 2.01 | 1 | 7 | 2 | 3 | 5 |
|  | pahc23-Sad | 20 | 0 | 2.55 | 1.61 | 1 | 7 | 2 | 2 | 3 |
|  | pahc24-Scared | 20 | 0 | 4.10 | 2.10 | 1 | 7 | 3 | 4 | 6 |
|  | pahc25-Insecure about the future | 20 | 0 | 4.80 | 1.67 | 1 | 7 | 4 | 5 | 6 |
|  | pahc26-Optimistic | 20 | 0 | 3.00 | 1.12 | 1 | 6 | 2 | 3 | 4 |
|  | pahc27-Calm | 20 | 0 | 3.00 | 1.41 | 1 | 6 | 2 | 3 | 4 |
|  | pahc28-Confident | 20 | 0 | 3.75 | 1.62 | 1 | 7 | 3 | 3 | 4 |
|  | pahc29- Able to handle the situation | 20 | 0 | 5.05 | 1.05 | 3 | 7 | 4 | 5 | 6 |

*Note*: SD: standard deviation; Min: minimum value; Max: maximum value; Q1 and Q3: first and third quartile, respectively.

### Table SM2.30 Crosstab Centre × I-PAHC30 - Having cancer (counts and row percentages)

| Centre | No | Yes | Total |
| --- | --- | --- | --- |
| Bologna | 19 | 34 | 53 |
|  | 35.85% | 64.15% |  |
| Genova | 113 | 30 | 143 |
|  | 79.02% | 20.98% |  |
| Lecce | 23 | 14 | 37 |
|  | 62.16% | 37.84% |  |
| Modena | 12 | 8 | 20 |
|  | 60.00% | 40.00% |  |
| Total | 167 | 86 | 253 |
|  | 66.01% | 33.99% |  |

### Table SM2.30a Descriptive statistics for the item I-PACH30 - If you have cancer now, how emotionally burdensome is it for you?

| Centre | Valid | Missing | Mean | SD | Min | Max | Q1 | Median | Q3 |
| --- | --- | --- | --- | --- | --- | --- | --- | --- | --- |
| Total | 86 | 0 | 5.09 | 1.66 | 1 | 7 | 4 | 5 | 7 |
| Bologna | 34 | 0 | 5.26 | 1.66 | 1 | 7 | 4 | 5 | 7 |
| Genova | 30 | 0 | 4.27 | 1.68 | 1 | 7 | 3 | 5 | 5 |
| Lecce | 14 | 0 | 5.93 | 1.07 | 4 | 7 | 5 | 6 | 7 |
| Modena | 8 | 0 | 6.00 | 1.07 | 4 | 7 | 6 | 6 | 7 |

*Note*: SD: standard deviation; Min: minimum value; Max: maximum value; Q1 and Q3: first and third quartile, respectively.

### Table SM2.31 Crosstab Centre × I-PAHC31 - Having had cancer (counts and row percentages)

| Centre | No | Yes | Total |
| --- | --- | --- | --- |
| Bologna | 23 | 29 | 52 |
|  | 44.23% | 55.77% |  |
| Genova | 55 | 93 | 148 |
|  | 37.16% | 62.84% |  |
| Lecce | 21 | 15 | 36 |
|  | 58.33% | 41.67% |  |
| Modena | 6 | 14 | 20 |
|  | 30.00% | 70.00% |  |
| Total | 105 | 151 | 256 |
|  | 41.02% | 58.98% |  |

### Table SM2.31a Descriptive statistics for the item I-PACH31 - If you had cancer in the past, how emotionally burdensome was it for you?

| Centre | Valid | Missing | Mean | SD | Min | Max | Q1 | Median | Q3 |
| --- | --- | --- | --- | --- | --- | --- | --- | --- | --- |
| Total | 151 | 0 | 5.19 | 1.72 | 1 | 7 | 4 | 5 | 7 |
| Bologna | 29 | 0 | 5.41 | 1.88 | 1 | 7 | 4 | 6 | 7 |
| Genova | 93 | 0 | 4.91 | 1.71 | 1 | 7 | 4 | 5 | 6 |
| Lecce | 15 | 0 | 5.87 | 1.73 | 3 | 7 | 5 | 7 | 7 |
| Modena | 14 | 0 | 5.86 | 1.03 | 4 | 7 | 5 | 6 | 7 |

*Note*: SD: standard deviation; Min: minimum value; Max: maximum value; Q1 and Q3: first and third quartile, respectively.

### Table SM2.32 Descriptive statistics for the item I-PACH32 - Are you worried about the chance of getting cancer (again)?

| Centre | Valid | Missing | Mean | SD | Min | Max | Q1 | Median | Q3 |
| --- | --- | --- | --- | --- | --- | --- | --- | --- | --- |
| Total | 263 | 8 | 4.95 | 1.76 | 1 | 7 | 4 | 5 | 7 |
| Bologna | 54 | 3 | 5.20 | 1.68 | 1 | 7 | 4 | 5 | 7 |
| Genova | 152 | 2 | 4.68 | 1.72 | 1 | 7 | 3 | 5 | 6 |
| Lecce | 37 | 3 | 5.27 | 2.04 | 1 | 7 | 4 | 6 | 7 |
| Modena | 20 | 0 | 5.70 | 1.34 | 2 | 7 | 5 | 6 | 7 |

*Note*: SD: standard deviation; Min: minimum value; Max: maximum value; Q1 and Q3: first and third quartile, respectively.

### Table SM2.33 Crosstab Centre × I-PACH33 - Having had family members with cancer (counts and row percentages)

| Centre | No | Yes | Total |
| --- | --- | --- | --- |
| Bologna | 9 | 45 | 54 |
|  | 16.67% | 83.33% |  |
| Genova | 17 | 135 | 152 |
|  | 11.18% | 88.82% |  |
| Lecce | 0 | 36 | 36 |
|  | 0.00% | 100.00% |  |
| Modena | 0 | 20 | 20 |
|  | 0.00% | 100.00% |  |
| Total | 26 | 236 | 262 |
|  | 9.92% | 90.08% |  |

### Table SM2.33a Descriptive statistics for the item I-PACH33 - If you have had family members with cancer, how did it impact on your life?

| Centre | Valid | Missing | Mean | SD | Min | Max | Q1 | Median | Q3 |
| --- | --- | --- | --- | --- | --- | --- | --- | --- | --- |
| Total | 236 | 0 | 5.72 | 1.69 | 1 | 7 | 5 | 7 | 7 |
| Bologna | 45 | 0 | 5.82 | 1.66 | 1 | 7 | 5 | 7 | 7 |
| Genova | 135 | 0 | 5.79 | 1.58 | 1 | 7 | 5 | 7 | 7 |
| Lecce | 36 | 0 | 6.19 | 1.33 | 2 | 7 | 6 | 7 | 7 |
| Modena | 20 | 0 | 4.15 | 2.23 | 1 | 7 | 2 | 4 | 6 |

*Note*: SD: standard deviation; Min: minimum value; Max: maximum value; Q1 and Q3: first and third quartile, respectively.

### Table SM2.33PSY5 Crosstab Centre × answer to the question "Would you like to talk to a psychologist in addition to the clinical geneticist about these issues?" in the section "Living with cancer" (counts and row percentages)

| Centre | No | Yes | Total |
| --- | --- | --- | --- |
| Bologna | 39 | 16 | 55 |
|  | 70.91% | 29.09% |  |
| Genova | 79 | 59 | 138 |
|  | 57.25% | 42.75% |  |
| Lecce | 21 | 14 | 35 |
|  | 60.00% | 40.00% |  |
| Modena | 6 | 14 | 20 |
|  | 30.00% | 70.00% |  |
| Total | 145 | 103 | 248 |
|  | 58.47% | 41.53% |  |

### Table SM2.33PSY5a Correlations between the items of the section "Living with cancer" and answer to the question "Would you like to talk to a psychologist in addition to the clinical geneticist about these issues?"

| Item | Total^§^ | Bologna | Genova | Lecce | Modena |
| --- | --- | --- | --- | --- | --- |
| pahc30a° | .03 | .20 | .10 | .20 | .56 |
| pahc30b^ | .02 | .06 | -.01 | .27 | NC |
| pahc31a° | .06 | .20 | .08 | .20 | .46 |
| pahc31b^ | .07 | .16 | .03 | -.19 | .35 |
| pahc32 | .12 | .15 | -.06 | .20 | .48* |
| pahc33a° | .06 | .08 | .18 | NC | NC |
| pahc33b^ | .05 | .10 | .11 | .01 | -.29 |

*Note*: §: corrected for the nesting of participants into centres. °: dichotomized variable (No/Yes), *phi* coefficient; ^: variable that takes into account only cases that answered "Yes" to the question and provided quantitative information; *Kendall's tau* coefficient; **: *p* <. 01; *: *p* <. 05; NC: not computable

# **SM 3 - Dimensionality and Item analysis of the I-PAHC scales**

## **SM 3.1 Hereditary predisposition scale**

The dimensionality of the questionnaire scales was evaluated using an exploratory factor analysis approach. We first investigated the optimal number of factors to be extracted using the scree-test (Cattell, 1966), parallel analysis (PA, Horn, 1965), and the Minimum Average Partial (MAP) correlation statistic (Velicer, 1976). The first method suggests that the optimal number of factors corresponds to the factors before which the downward curve of the eigenvalues seems to flatten out. PA compares the observed eigenvalues to the eigenvalues generated from a simulated matrix of random data of the same size. On the basis of the recommendations of Buja and Eyuboglu (1992), we performed PA on 1,000 random correlation matrices obtained through permutation of the raw data, and following Longman et al. (1989) we considered the 95th percentile eigenvalues as the threshold values. The last method indicates that the optimal number of factors is the one at which the average partial correlation of the variables, after partialling out the factors, reaches its minimum.

In the case of the Hereditary predisposition scale, all these methods converged to suggest that the optimal number of factors was one (Figure SM3.1)


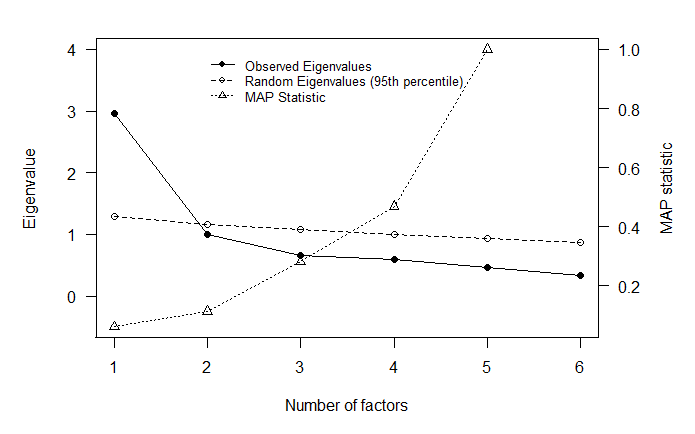


#### Figure SM3.1 Dimensionality analysis of the items of the section Hereditary predisposition

We then performed an exploratory factor analysis on the within correlation matrix (see main text) setting to one the number of factors to be extracted (extraction method: minimum residual (Comrey and Lee, 1992). To be considered as adequate, factor loadings should exceed .30 and are reported in Table SM3.1.

We also carried out an item analysis in order to investigate the discrimination of the items (i.e., their ability to distinguish participants with higher score on the scale from those with lower scores) as indexed by the corrected item-total correlation. Following Nunnally and Bernestein (1994) we considered as indices of adequate discrimination values larger than .30. We then considered squared multiple correlation (SMC), which is a measure of the proportion of variance of an item that is shared with the other items, and an estimate of the variance accounted for by the common factors. Items with a SMC smaller than .10 are thus unlikely to substantially contribute to the measurement model, and can be removed from the item pool (Tabachnick and Fidell, 1996). We then considered the value of alpha if the item were removed from the scale: since the Spearman-Brown (Brown, 1910; Spearman, 1910) prophecy states that the reliability increases as the number of item increases, if the removal of one item led to an increase of at least .01 of the reliability of the scale (indexed as Cronbach's alpha, Cronbach, 1951), then it can be assumed that the item does not contribute to the accuracy of the measure. Reliability was computed as internal consistency with the Cronbach's alpha and as an estimate of the proportion of variance in the unit-weighted total score attributable to all sources of true score variance with the Omega coefficient (McDonald, 1999; Reise et al., 2013). Reliability values larger than .70 are usually considered as adequate (Nunnally and Bernstein, 1994).

### Table SM3.1 Item analysis of the items in the section Hereditary predisposition

| Item | *r_cit_* | SMC | α_w/o_ | Reliability | FL |
| --- | --- | --- | --- | --- | --- |
| pahc3 | .51 | .40 | .76 | .79 - .86 | .74 |
| pahc4 | .64 | .48 | .73 |  | .72 |
| pahc5 | .41 | .21 | .79 |  | .38 |
| pahc6 | .43 | .24 | .78 |  | .58 |
| pahc7 | .68 | .49 | .72 |  | .53 |
| pahc8 | .57 | .37 | .75 |  | .74 |

*Note*: *r_cit_*: corrected item-total correlation; SMC: squared multiple correlation; α_w/o_: alpha without the item statistic; coefficients in the Reliability column are Cronbach's Alpha and Omega; FL: factor loading on the single factor.

### Table SM3.2 Descriptive statistics of the scale score in the section Hereditary predisposition

| Centre | N | Mean | SD | Min | Max | Q1 | Median | Q3 | *r*_Psy_ |
| --- | --- | --- | --- | --- | --- | --- | --- | --- | --- |
| Total | 267 | 27.83 | 7.53 | 6 | 42 | 23 | 28 | 34 | .18 |
| Bologna | 57 | 28.22 | 7.71 | 9 | 42 | 23 | 29 | 34 | .21 |
| Genova | 153 | 27.38 | 7.45 | 9 | 42 | 23 | 28 | 33 | .12 |
| Lecce | 37 | 31.06 | 7.29 | 6 | 42 | 28 | 32 | 36 | .33 |
| Modena | 20 | 24.20 | 6.07 | 16 | 36 | 20 | 23 | 28 | .32 |

*Note*: SD: standard deviation; Min: minimum value; Max: maximum value; Q1 and Q3: first and third quartile, respectively; *r*_Psy_: correlation with the question "Would you like to speak with a psychosocial worker in addition to the clinical geneticist/genetic counselor about these issues?" of this section.

## **SM 3.2 Family and social environment**

The dimensionality analyses supported the unidimensionality of the scale (Figure SM3.2), and all other statistics met the criteria described above.


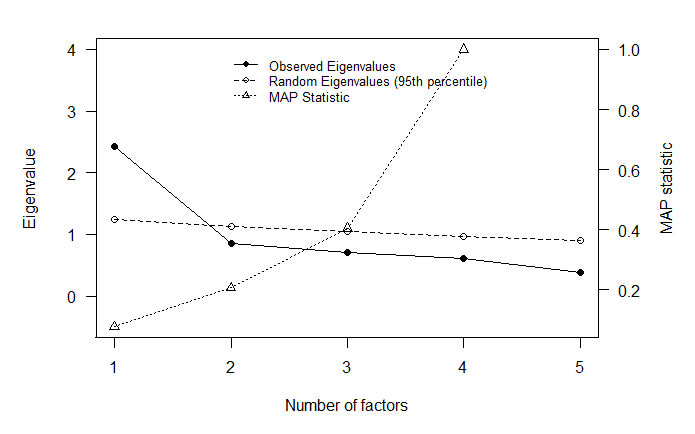


#### Figure SM3.2 Dimensionality analysis of the items of the section Family and social environment

### Table SM3.3 Item analysis of the items in the section Family and social environment

| Item | *r_cit_* | SMC | α_w/o_ | Reliability | FL |
| --- | --- | --- | --- | --- | --- |
| pahc9 | .58 | .42 | .64 | .73 - .78 | .74 |
| pahc10 | .57 | .41 | .65 |  | .72 |
| pahc11 | .33 | .12 | .73 |  | .38 |
| pahc12 | .50 | .25 | .67 |  | .58 |
| pahc13 | .46 | .21 | .69 |  | .53 |

*Note*: *r_cit_*: corrected item-total correlation; SMC: squared multiple correlation; α_w/o_: alpha without the item statistic; coefficients in the Reliability column are Cronbach's Alpha and Omega; FL: factor loading on the single factor

### Table SM3.4 Descriptive statistics of the scale score in the section Family and social environment

| Centre | N | Mean | SD | Min | Max | Q1 | Median | Q3 | *r*_Psy_ |
| --- | --- | --- | --- | --- | --- | --- | --- | --- | --- |
| Total | 267 | 19.66 | 7.15 | 5 | 35 | 14 | 20 | 25 | .22 |
| Bologna | 57 | 19.53 | 7.77 | 5 | 35 | 14 | 20 | 25 | .54 |
| Genova | 153 | 19.06 | 7.00 | 5 | 35 | 14 | 19 | 24 | .09 |
| Lecce | 37 | 23.70 | 6.12 | 11 | 34 | 20 | 24 | 28 | .12 |
| Modena | 20 | 17.15 | 5.79 | 9 | 27 | 12 | 18 | 21 | .44 |

*Note*: SD: standard deviation; Min: minimum value; Max: maximum value; Q1 and Q3: first and third quartile, respectively; *r*_Psy_: correlation with the question "Would you like to speak with a psychosocial worker in addition to the clinical geneticist/genetic counselor about these issues?" of this section.

## **SM 3.3 Children**

The dimensionality analyses supported the unidimensionality of the scale (Figure SM3.3), and all other statistics met the criteria described above.


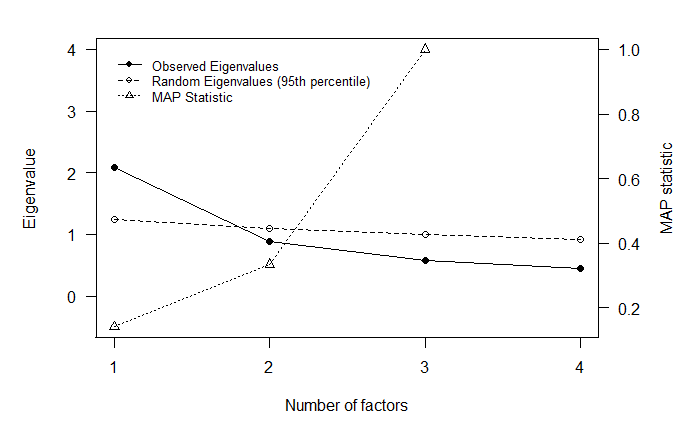


#### Figure SM3.3 Dimensionality analysis of the items of the section Children

### Table SM3.5 Item analysis of the items in the section Children

| Item | *rcit* | SMC | α_w/o_ | Reliability | FL |
| --- | --- | --- | --- | --- | --- |
| pahc15b | .60 | .39 | .58 | .71 - .77 | .75 |
| pahc16 | .46 | .25 | .66 |  | .56 |
| pahc17 | .47 | .25 | .66 |  | .54 |
| pahc18 | .45 | .31 | .67 |  | .57 |

*Note*: *r_cit_*: corrected item-total correlation; SMC: squared multiple correlation; α_w/o_: alpha without the item statistic; coefficients in the Reliability column are Cronbach's Alpha and Omega; FL: factor loading on the single factor

### Table SM3.6 Descriptive statistics of the scale score in the section Children

| Centre | N | Mean | SD | Min | Max | Q1 | Median | Q3 | *r*_Psy_ |
| --- | --- | --- | --- | --- | --- | --- | --- | --- | --- |
| Total | 206 | 17.41 | 6.10 | 4 | 28 | 12 | 18 | 22 | .24 |
| Bologna | 48 | 17.56 | 6.42 | 4 | 28 | 12 | 18 | 22 | .19 |
| Genova | 111 | 16.85 | 6.16 | 4 | 28 | 11 | 17 | 21 | .28 |
| Lecce | 29 | 18.87 | 5.64 | 7 | 28 | 15 | 20 | 23 | .24 |
| Modena | 18 | 18.15 | 5.52 | 11 | 28 | 14 | 17 | 23 | .11 |

*Note*: SD: standard deviation; Min: minimum value; Max: maximum value; Q1 and Q3: first and third quartile, respectively; *r*_Psy_: correlation with the question "Would you like to speak with a psychosocial worker in addition to the clinical geneticist/genetic counselor about these issues?" of this section.

## **SM 3.4 Emotions with respect to genetic counseling and testing**

The dimensionality analyses supported the expected two-factor structure (Figure SM3.4)


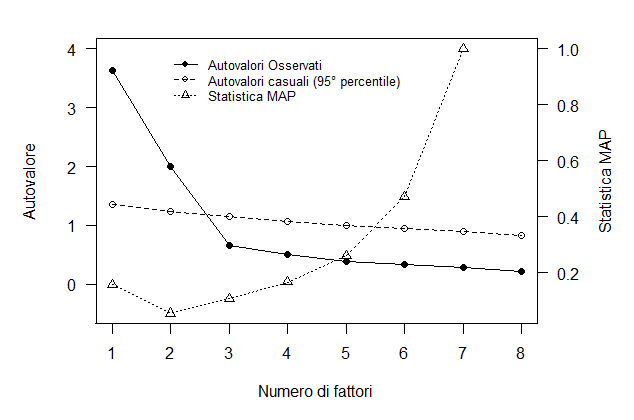


#### Figure SM3.4 Dimensionality analysis of the items of the section Emotions with respect to genetic counseling and testing (items I-PAHC22_I-PAHC29)

We then carried out an exploratory factor analysis setting to two the number of factors to be extracted (Promax rotation). All items had a substantial loading only on the expected factor (Table SM3.7).

### Table SM3.7 Factor loadings and factor correlation from the exploratory factor analysis on the items in the *Emotions with respect to genetic counseling and testing*

| Item | F1 | F2 |
| --- | --- | --- |
| pahc22-Tense | **.80** | .05 |
| pahc23-Sad | **.68** | -.05 |
| pahc24-Scared | **.93** | .05 |
| pahc25-Insecure about the future | **.79** | -.01 |
| pahc26-Optimistic | .12 | **.82** |
| pahc27-Calm | -.17 | **.77** |
| pahc28-Confident | .11 | **.86** |
| pahc29-Able to handle the situation | -.08 | **.53** |
| *r* with F1 |  | −.35 |

*Note*: Bolded loadings are those larger than |.30|

### Table SM3.8 Item analysis for the section Emotions with respect to genetic counseling and testing - Negative Affect

| Item | *r_cit_* | SMC | α_w/o_ | Reliability |
| --- | --- | --- | --- | --- |
| pahc22-Tense | .71 | .55 | .84 | .87 - .89 |
| pahc23-Sad | .65 | .43 | .87 |  |
| pahc24-Scared | .82 | .69 | .80 |  |
| pahc25-Insecure about the future | .73 | .57 | .84 |  |

*Note*: *r_cit_*: corrected item-total correlation; SMC: squared multiple correlation; α_w/o_: alpha without the item statistic; coefficients in the Reliability column are Cronbach's Alpha and Omega.

### Table SM3.9 Item analysis of the items in the section Emotions with respect to genetic counseling and testing - Positive Affect

| Item | *rcit* | SMC | α_s_ | Reliability |
| --- | --- | --- | --- | --- |
| pahc26-Optimistic | .68 | .51 | .77 | .83 - .85 |
| pahc27-Calm | .72 | .53 | .76 |  |
| pahc28-Confident | .72 | .53 | .75 |  |
| pahc29-Able to handle the situation | .51 | .27 | .83 |  |

*Note*: *r_cit_*: corrected item-total correlation; SMC: squared multiple correlation; α_w/o_: alpha without the item statistic; coefficients in the Reliability column are Cronbach's Alpha and Omega.

### Table SM3.10 Descriptive statistics for the scale Negative Affect

| Centre | Valid | Mean | SD | Min | Max | Q1 | Median | Q3 | *r*_Psy_ |
| --- | --- | --- | --- | --- | --- | --- | --- | --- | --- |
| Total | 254 | 13.52 | 6.65 | 4 | 28 | 8 | 12 | 19 | .25 |
| Bologna | 53 | 13.85 | 7.18 | 4 | 28 | 8 | 15 | 20 | .37 |
| Genova | 145 | 12.37 | 6.19 | 4 | 28 | 8 | 11 | 16 | .08 |
| Lecce | 36 | 16.83 | 6.95 | 4 | 28 | 12 | 18 | 23 | .57 |
| Modena | 20 | 15.00 | 5.93 | 4 | 28 | 11 | 14 | 19 | .40 |

*Note*: SD: standard deviation; Min: minimum value; Max: maximum value; Q1 and Q3: first and third quartile, respectively; *r*_Psy_: correlation with the question "Would you like to speak with a psychosocial worker in addition to the clinical geneticist/genetic counselor about these issues?" of this section.

### Table SM3.11 Descriptive statistics for the scale Positive Affect

| Centre | Valid | Mean | SD | Min | Max | Q1 | Median | Q3 | *r*_Psy_ |
| --- | --- | --- | --- | --- | --- | --- | --- | --- | --- |
| Total | 266 | 18.37 | 5.38 | 4 | 28 | 15 | 18 | 23 | -.13 |
| Bologna | 56 | 19.13 | 5.58 | 7 | 28 | 16 | 19 | 24 | -.16 |
| Genova | 153 | 18.58 | 5.24 | 5 | 28 | 15 | 18 | 23 | -.11 |
| Lecce | 37 | 18.30 | 5.51 | 4 | 28 | 16 | 19 | 21 | .01 |
| Modena | 20 | 14.80 | 4.44 | 7 | 24 | 12 | 14 | 18 | -.46 |

*Note*: SD: standard deviation; Min: minimum value; Max: maximum value; Q1 and Q3: first and third quartile, respectively; *r*_Psy_: correlation with the question "Would you like to speak with a psychosocial worker in addition to the clinical geneticist/genetic counselor about these issues?" of this section.

# **SM 4 - Descriptive statistics of the psychological tests**

### Table SM4.1 Descriptive statistics for the scale *Distress Thermometer*

| Centre | Valid | Missing | Mean | SD | Min | Max | Q1 | Median | Q3 |
| --- | --- | --- | --- | --- | --- | --- | --- | --- | --- |
| Total | 242 | 29 | 4.32 | 3.02 | 0 | 10 | 2 | 4 | 7 |
| Bologna | 45 | 12 | 4.36 | 3.34 | 0 | 10 | 0 | 5 | 7 |
| Genova | 145 | 9 | 4.45 | 2.97 | 0 | 10 | 2 | 4 | 7 |
| Lecce | 32 | 8 | 3.00 | 2.63 | 0 | 10 | 1 | 3 | 5 |
| Modena | 20 | 0 | 5.45 | 2.68 | 1 | 10 | 4 | 6 | 7 |

*Note*: SD: standard deviation; Min: minimum value; Max: maximum value; Q1 and Q3: first and third quartile, respectively

### Table SM4.2 Descriptive statistics for the scale *Practical problems*

| Centre | Valid | Missing | Mean | SD | Min | Max | Q1 | Median | Q3 | α |
| --- | --- | --- | --- | --- | --- | --- | --- | --- | --- | --- |
| Total | 262 | 9 | 0.66 | 1.01 | 0 | 5 | 0 | 0 | 1 | .40 |
| Bologna | 55 | 2 | 0.55 | 1.17 | 0 | 5 | 0 | 0 | 1 | .65 |
| Genova | 152 | 2 | 0.82 | 1.03 | 0 | 5 | 0 | 1 | 1 | .32 |
| Lecce | 35 | 5 | 0.40 | 0.69 | 0 | 2 | 0 | 0 | 1 | .36 |
| Modena | 20 | 0 | 0.20 | 0.52 | 0 | 2 | 0 | 0 | 0 | .65 |

*Note*: SD: standard deviation; Min: minimum value; Max: maximum value; Q1 and Q3: first and third quartile, respectively; α: Cronbach's alpha

### Table SM4.3 Descriptive statistics for the scale *Family-related problems*

| Centre | Valid | Missing | Mean | SD | Min | Max | Q1 | Median | Q3 | α |
| --- | --- | --- | --- | --- | --- | --- | --- | --- | --- | --- |
| Total | 262 | 9 | 0.47 | 0.80 | 0 | 3 | 0 | 0 | 1 | .46 |
| Bologna | 55 | 2 | 0.44 | 0.79 | 0 | 3 | 0 | 0 | 1 | .57 |
| Genova | 152 | 2 | 0.58 | 0.88 | 0 | 3 | 0 | 0 | 1 | .44 |
| Lecce | 35 | 5 | 0.20 | 0.47 | 0 | 2 | 0 | 0 | 0 | .24 |
| Modena | 20 | 0 | 0.15 | 0.37 | 0 | 1 | 0 | 0 | 0 | NC |

*Note*: SD: standard deviation; Min: minimum value; Max: maximum value; Q1 and Q3: first and third quartile, respectively; α: Cronbach's alpha

### Table SM4.4 Descriptive statistics for the scale *Emotional problems*

| Centre | Valid | Missing | Mean | SD | Min | Max | Q1 | Median | Q3 | α |
| --- | --- | --- | --- | --- | --- | --- | --- | --- | --- | --- |
| Total | 263 | 8 | 2.38 | 1.75 | 0 | 6 | 1 | 2 | 3 | .67 |
| Bologna | 55 | 2 | 2.58 | 1.86 | 0 | 6 | 1 | 3 | 4 | .72 |
| Genova | 153 | 1 | 2.35 | 1.65 | 0 | 6 | 1 | 2 | 3 | .58 |
| Lecce | 35 | 5 | 1.89 | 1.88 | 0 | 6 | 0 | 2 | 4 | .81 |
| Modena | 20 | 0 | 2.90 | 1.89 | 0 | 6 | 1 | 3 | 4 | .77 |

*Note*: SD: standard deviation; Min: minimum value; Max: maximum value; Q1 and Q3: first and third quartile, respectively; α: Cronbach's alpha

### Table SM4.5 Descriptive statistics for the scale *Physical problems*

| Centre | Valid | Missing | Mean | SD | Min | Max | Q1 | Median | Q3 | α |
| --- | --- | --- | --- | --- | --- | --- | --- | --- | --- | --- |
| Total | 262 | 9 | 4.11 | 3.44 | 0 | 21 | 2 | 4 | 6 | .80 |
| Bologna | 54 | 3 | 4.22 | 3.88 | 0 | 21 | 2 | 4 | 6 | .85 |
| Genova | 153 | 1 | 4.20 | 3.30 | 0 | 17 | 2 | 4 | 6 | .75 |
| Lecce | 35 | 5 | 3.26 | 3.58 | 0 | 15 | 0 | 3 | 5 | .85 |
| Modena | 20 | 0 | 4.60 | 2.93 | 0 | 14 | 3 | 5 | 6 | .73 |

*Note*: SD: standard deviation; Min: minimum value; Max: maximum value; Q1 and Q3: first and third quartile, respectively; α: Cronbach's alpha

### Table SM4.6 Crosstab Centre × Question about having had spiritual/religious problems (counts and row percentages)

| Centre | No | Yes | Total |
| --- | --- | --- | --- |
| Bologna | 48 | 5 | 53 |
|  | 90.57% | 9.43% |  |
| Genova | 137 | 14 | 151 |
|  | 90.73% | 9.27% |  |
| Lecce | 31 | 4 | 35 |
|  | 88.57% | 11.43% |  |
| Modena | 20 | 0 | 20 |
|  | 100.00% | 0.00% |  |
| Total | 236 | 23 | 259 |
|  | 91.12% | 8.88% |  |

### Table SM4.7 Descriptive statistics for the scale *CES-D*

| Centre | Valid | Missing | Mean | SD | Min | Max | Q1 | Median | Q3 | α |
| --- | --- | --- | --- | --- | --- | --- | --- | --- | --- | --- |
| Total | 262 | 9 | 18.35 | 5.42 | 10 | 33 | 14 | 18 | 22 | .77 |
| Bologna | 56 | 1 | 17.96 | 5.55 | 10 | 32 | 14 | 18 | 21 | .78 |
| Genova | 152 | 2 | 18.29 | 5.34 | 10 | 33 | 14 | 18 | 22 | .75 |
| Lecce | 34 | 6 | 18.24 | 5.67 | 10 | 32 | 14 | 17 | 23 | .81 |
| Modena | 20 | 0 | 20.15 | 5.34 | 11 | 32 | 16 | 20 | 23 | .79 |

*Note*: SD: standard deviation; Min: minimum value; Max: maximum value; Q1 and Q3: first and third quartile, respectively; α: Cronbach's alpha

### Table SM4.8 Descriptive statistics for the scale *STAI-X3*

| Centre | Valid | Missing | Mean | SD | Min | Max | Q1 | Median | Q3 | α |
| --- | --- | --- | --- | --- | --- | --- | --- | --- | --- | --- |
| Total | 265 | 6 | 20.03 | 6.28 | 10 | 40 | 15 | 18 | 24 | .90 |
| Bologna | 56 | 1 | 19.77 | 5.86 | 10 | 33 | 16 | 18 | 24 | .90 |
| Genova | 153 | 1 | 18.59 | 5.49 | 10 | 38 | 15 | 17 | 22 | .88 |
| Lecce | 36 | 4 | 22.69 | 6.07 | 11 | 36 | 18 | 23 | 28 | .92 |
| Modena | 20 | 0 | 26.95 | 7.67 | 14 | 40 | 21 | 28 | 32 | .93 |

*Note*: SD: standard deviation; Min: minimum value; Max: maximum value; Q1 and Q3: first and third quartile, respectively; α: Cronbach's alpha

### Table SM4.9 Descriptive statistics for the scale *PSWQ*

| Centre | Valid | Missing | Mean | SD | Min | Max | Q1 | Median | Q3 | α |
| --- | --- | --- | --- | --- | --- | --- | --- | --- | --- | --- |
| Total | 260 | 11 | 47.96 | 12.98 | 20 | 77 | 38 | 47 | 58 | .91 |
| Bologna | 54 | 3 | 47.72 | 12.42 | 20 | 76 | 38 | 47 | 58 | .88 |
| Genova | 150 | 4 | 47.66 | 13.67 | 20 | 77 | 37 | 47 | 58 | .92 |
| Lecce | 36 | 4 | 48.61 | 11.25 | 22 | 74 | 42 | 51 | 56 | .89 |
| Modena | 20 | 0 | 49.70 | 12.83 | 31 | 74 | 41 | 47 | 60 | .94 |

*Note*: SD: standard deviation; Min: minimum value; Max: maximum value; Q1 and Q3: first and third quartile, respectively; α: Cronbach's alpha

# **SM 5 - Correlations of I-PAHC scores with sociodemographic and psychological variables.**

### Table SM5.1 Correlations of I-PACH scores with the sociodemographic variables. (part 1 of 2)

| I-PAHC variable | Age | Sex° | Educ^ | Single | Coliving | Married | Divorced | Widow |
| --- | --- | --- | --- | --- | --- | --- | --- | --- |
| I-PAHC1 - motivation | .03 | -.03 | .08 | -.06 | .13 | .00 | .03 | -.11 |
| I-PAHC2 – no advice from specialist | .08 | .18 | .03 | -.10 | -.06 | .21 | -.12 | -.06 |
| I-PAHC2 – useful advice from specialist | -.05 | -.19 | -.04 | .08 | .09 | -.21 | .10 | .09 |
| I-PAHC2 – useless advice from specialist | -.08 | .02 | .01 | .03 | -.06 | .01 | .04 | -.06 |
| Hereditary Predisposition | **-.24** | -.15 | -.01 | .03 | .08 | -.08 | .05 | -.04 |
| Family and social relationships | -.08 | -.05 | -.04 | .01 | .01 | -.03 | .06 | -.03 |
| Children | **-.26** | -.04 | -.06 | -.01 | **.23** | -.13 | .02 | .00 |
| I-PAHC19b support from partner | -.09 | .12 | .01 | -.06 | .01 | .03 | -.03 | .07 |
| I-PAHC20b - support from family | -.07 | .13 | -.03 | .00 | .03 | -.03 | -.08 | .13 |
| I-PAHC21b - support from friends | **-.26** | -.15 | .08 | .07 | .05 | -.11 | -.02 | .09 |
| Negative Emotions | **-.24** | -.18 | -.01 | .05 | .05 | -.02 | .03 | -.12 |
| Positive Emotions | .06 | .03 | -.09 | -.09 | .09 | -.04 | .03 | .06 |
| I-PAHC30 - emotional burden current cancer | .05 | -.12 | .02 | -.03 | .01 | .02 | -.02 | .01 |
| I-PAHC31 - emotional burden past cancer | .16 | -.16 | -.13 | -.10 | .02 | .15 | -.13 | -.03 |
| I-PAHC32 - worry for cancer | -.19 | -.11 | -.01 | .03 | .04 | -.02 | .00 | -.04 |
| I-PAHC33 - impact of a beloved one's cancer | -.10 | -.03 | -.05 | .01 | -.05 | -.03 | .04 | .07 |

*Note*: Bolded coefficients are statistically different from zero at *p* = .001 (see text); °: female = 0, male = 1; Educ: education; ^: this variable recoded as years of education;

### Table SM5.1 Correlations of I-PACH scores with the sociodemographic variables. (part 2 of 2)

| I-PAHC variable | unempl | empl | self-empl | retired | housew | student | children | current | past |
| --- | --- | --- | --- | --- | --- | --- | --- | --- | --- |
| I-PAHC1 - motivation | .12 | .05 | .01 | -.01 | -.12 | -.09 | .02 | .11 | -.01 |
| I-PAHC2 – no advice from specialist | -.07 | -.06 | .12 | -.01 | -.03 | .11 | .03 | -.04 | .01 |
| I-PAHC2 – useful advice from specialist | .06 | .02 | -.08 | .05 | .03 | -.15 | -.02 | .07 | .02 |
| I-PAHC2 – useless advice from specialist | .03 | .09 | -.09 | -.10 | -.01 | .09 | -.02 | -.07 | -.07 |
| Hereditary Predisposition | .06 | .13 | -.08 | -.19 | .06 | -.01 | -.08 | .00 | -.06 |
| Family and social relationships | .01 | .03 | -.01 | .00 | -.02 | -.06 | .03 | -.02 | -.02 |
| Children | .09 | .08 | -.10 | -.08 | .02 | **NC** | .15 | -.05 | -.01 |
| I-PAHC19b support from partner | .03 | -.01 | -.01 | -.02 | .03 | -.03 | -.06 | -.12 | .09 |
| I-PAHC20b - support from family | .00 | -.03 | -.02 | -.08 | .12 | .07 | .11 | .02 | -.05 |
| I-PAHC21b - support from friends | .09 | .10 | -.05 | **-.24** | .08 | .08 | -.05 | .09 | -.17 |
| Negative Emotions | .10 | .14 | -.13 | -.16 | .00 | .04 | -.13 | -.03 | .03 |
| Positive Emotions | .07 | -.04 | -.04 | .06 | .04 | -.10 | .04 | .02 | .07 |
| I-PAHC30 - emotional burden current cancer | .05 | .10 | -.12 | -.01 | -.01 | -.08 | -.01 | **.63** | -.12 |
| I-PAHC31 - emotional burden past cancer | -.02 | -.10 | -.04 | .09 | .18 | -.16 | .10 | -.15 | **.65** |
| I-PAHC32 - worry for cancer | .03 | .05 | .00 | -.12 | .04 | .00 | -.07 | -.03 | .10 |
| I-PAHC33 - impact of a beloved one's cancer | .09 | -.03 | .00 | -.13 | .09 | .08 | -.02 | -.13 | -.11 |

*Note*: Bolded coefficients are statistically different from zero at *p* = .001 (see text); unempl: unemployed; empl; employee; self-empl: self-employed; housew: housewife; current: currently having a cancer; past: having had a cancer in the past. NC: not computableR

### Table SM5.2 Correlations of I-PACH scores with the other psychological measures.

| I-PAHC variable | DT | Prac | Fam | Emo | Phys | Spir | STAI-X3 | CES-D | PSWQ |
| --- | --- | --- | --- | --- | --- | --- | --- | --- | --- |
| I-PAHC1 - motivation | .04 | .10 | .06 | .07 | .18 | .00 | -.10 | .02 | .01 |
| I-PAHC2 – no advice from specialist | -.09 | -.04 | -.03 | -.09 | -.02 | -.05 | -.16 | -.02 | -.05 |
| I-PAHC2 – useful advice from specialist | .00 | .02 | -.01 | .08 | .01 | .01 | .13 | -.02 | .03 |
| I-PAHC2 – useless advice from specialist | **.20** | .04 | .08 | .02 | .02 | .09 | .06 | .09 | .04 |
| Hereditary Predisposition | **.27** | .04 | .10 | **.38** | .15 | .08 | **.39** | **.26** | **.39** |
| Family and social relationships | .19 | .01 | .10 | .20 | .10 | .03 | **.33** | **.21** | **.26** |
| Children | **.28** | .16 | .18 | **.28** | .20 | -.02 | **.34** | **.36** | **.35** |
| I-PAHC19b support from partner | .03 | -.19 | **-.25** | -.01 | -.06 | -.02 | .03 | -.12 | -.08 |
| I-PAHC20b - support from family | -.03 | -.13 | -.16 | .07 | -.11 | -.12 | -.03 | **-.21** | -.11 |
| I-PAHC21b - support from friends | .12 | -.10 | -.08 | .03 | -.07 | .08 | .16 | -.10 | -.03 |
| Negative Emotions | **.41** | .07 | .14 | **.50** | **.28** | .06 | **.64** | **.39** | **.37** |
| Positive Emotions | **-.21** | -.06 | -.09 | **-.24** | -.06 | -.03 | **-.27** | **-.26** | -.19 |
| I-PAHC30 - emotional burden current cancer | .11 | -.04 | -.05 | .16 | **.22** | .09 | **.23** | **.23** | .04 |
| I-PAHC31 - emotional burden past cancer | .06 | -.02 | .05 | -.01 | .06 | .02 | -.01 | .05 | .09 |
| I-PAHC32 - worry for cancer | **.29** | .12 | .12 | **.27** | .20 | .14 | **.29** | **.32** | **.34** |
| I-PAHC33 - impact of a beloved one's cancer | .12 | .07 | .00 | .14 | .00 | .03 | .13 | .09 | .13 |

*Note*: Bolded coefficients are statistically different from zero at *p* = .001 (see text); DT: Distress Thermometer; Prac: Practical Problems; Fam: Family-related problems; Emo: emotional problems; Phys: physical problems, Spir: spiritual problems; CES-D: Center for Epidemiological Studies Depression Scale-Short Form; STAI-X3: State Anxiety Inventory-X3; PSWQ: Penn State Worry Questionnaire

# **SM 6 References**

Behling, O., and Law, K. S. (2000). *Translating questionnaires and other research instruments: Problems and solutions*. Thousand Oaks, CA: Sage.

Brown, W. (1910). Some experimental results in the correlation of mental abilities. *Br. J. Psychol. 1904-1920* 3, 296–322. doi:10.1111/j.2044-8295.1910.tb00207.x.

Buja, A., and Eyuboglu, N. (1992). Remarks on parallel analysis. *Multivariate Behav. Res.* 27, 509–540. doi:10.1207/s15327906mbr2704_2.

Cattell, R. B. (1966). The scree test for the number of factors. *Multivariate Behav. Res.* 1, 245–276. doi:10.1207/s15327906mbr0102_10.

Comrey, A. L., and Lee, H. B. (1992). *A first course in factor analysis*. 2nd ed. New York: Lawrence Erlbaum Associates Publishers.

Cronbach, L. J. (1951). Coefficient alpha and the internal structure of tests. *Psychometrika* 16, 297–334. doi:10.1007/BF02310555.

Horn, J. L. (1965). A rationale and test for the number of factors in factor analysis. *Psychometrika* 30, 179–185. doi:10.1007/BF02289447.

Longman, R. S., Cota, A. A., Holden, R. R., and Fekken, G. C. (1989). A regression equation for the parallel analysis criterion in principal components analysis: Mean and 95th percentile eigenvalues. *Multivariate Behav. Res.* 24, 59–69. doi:10.1207/s15327906mbr2401_4.

McDonald, R. P. (1999). *Test theory: A unified treatment*. New York: Lawrence Erlbaum Associates Publishers.

Nunnally, J. C., and Bernstein, I. H. (1994). *Psychometric theory*. 3rd ed. New York: McGraw-Hill.

Reise, S. P., Bonifay, W. E., and Haviland, M. G. (2013). Scoring and modeling psychological measures in the presence of multidimensionality. *J. Pers. Assess.* 95, 129–140. doi:10.1080/00223891.2012.725437.

Spearman, C. (1910). Correlation calculated from faulty data. *Br. J. Psychol.* 3, 271–295. doi:10.1111/j.2044-8295.1910.tb00206.x.

Tabachnick, B. G., and Fidell, L. S. . (1996). *Using multivariate statistics*. 3rd ed. New York: Harper Collins.

Velicer, W. (1976). Determining the number of components from the matrix of partial correlations. *Psychometrika* 41, 321–327. doi:10.1007/BF02293557.
